# ELK1 Uses Different DNA Binding Modes to Regulate Functionally Distinct Classes of Target Genes

**DOI:** 10.1371/journal.pgen.1002694

**Published:** 2012-05-10

**Authors:** Zaneta Odrowaz, Andrew D. Sharrocks

**Affiliations:** Faculty of Life Sciences, University of Manchester, Manchester, United Kingdom; Institut de Biologie de Lille, France

## Abstract

Eukaryotic transcription factors are grouped into families and, due to their similar DNA binding domains, often have the potential to bind to the same genomic regions. This can lead to redundancy at the level of DNA binding, and mechanisms are required to generate specific functional outcomes that enable distinct gene expression programmes to be controlled by a particular transcription factor. Here we used ChIP–seq to uncover two distinct binding modes for the ETS transcription factor ELK1. In one mode, other ETS transcription factors can bind regulatory regions in a redundant fashion; in the second, ELK1 binds in a unique fashion to another set of genomic targets. Each binding mode is associated with different binding site features and also distinct regulatory outcomes. Furthermore, the type of binding mode also determines the control of functionally distinct subclasses of genes and hence the phenotypic response elicited. This is demonstrated for the unique binding mode where a novel role for ELK1 in controlling cell migration is revealed. We have therefore uncovered an unexpected link between the type of binding mode employed by a transcription factor, the subsequent gene regulatory mechanisms used, and the functional categories of target genes controlled.

## Author Summary

One of the major outstanding questions in eukaryotic gene regulation is how transcription factors with seemingly very similar DNA binding specificities elicit specific biological responses. The ETS transcription factor family provides a paradigm for investigating this phenomenon. Here, we have focused on the ETS transcription factor ELK1, and by combining genome-wide binding analysis coupled with gene expression analysis we have dissected two distinct gene regulatory activities for this transcription factor. In each of these regulatory modes, ELK1 exhibits distinct DNA binding characteristics which correlate with either positive or negative transcriptional activities and give rise to functionally distinct gene expression programmes. We demonstrate a novel function for ELK1 in controlling cell migration through one of these regulatory modes. Thus, we have demonstrated a clear link between the types of regulatory region bound by a transcription factor and its ability to control gene expression (i.e. in a positive or negative manner) and the functional downstream consequences of its target gene cohort. This work has implications for understanding how members of other multi-protein transcription factor families might function to generate different downstream functional consequences through engaging with different types of regulatory regions.

## Introduction

Eukaryotic transcription factors are grouped into families based on their common DNA binding domains and these families can extend to dozens of members in mammalian cells. This is typified by the ETS-domain containing transcription factors, with human cells encoding 28 different proteins (reviewed in [1]). All these transcription factors contain very similar DNA-binding domains, and at least *in vitro*, their DNA binding specificities are very similar [2]. It is therefore a challenge to understand how individual family members can control specific gene expression programmes without unwanted crosstalk. Such crosstalk would potentially lead to functional redundancy due to the ability of different family members to bind to the same sites. Indeed, this has been suggested by genome-wide interrogation of DNA binding by different ETS transcription factors, where multiple family members can associate with the same genomic regions [3]–[5]. Such functional redundancy might explain why many of the phenotypes caused by the loss of ETS proteins are relatively subtle, despite the widespread expression of these transcription factors (reviewed in [6]). However, even with this huge potential for functional redundancy, several mouse knockout studies have revealed specific phenotypes for individual ETS transcription factors, suggesting that they function at least partially in a non-redundant fashion. One way for helping to achieve this specificity of action is through functional cooperation with other transcription factors. This is illustrated by the association of ETS1 with RUNX1 [4] and ELK1 with SRF [5], although in both cases, this co-association is only extended to a minority of the binding events identified *in vivo*.

ELK1 together with ELK4/SAP-1 and ELK3/SAP-2/Net, constitute the ternary complex factor (TCF) subfamily of ETS-domain transcription factors (reviewed in [7]–[8]). Like all ETS proteins, these transcription factors all bind to variants of the GGAA/T motif embedded in a larger 10 bp consensus sequence *in vitro*, and in the case of ELK1, this binding preference is also recapitulated *in vivo* [5]. Members of the TCF subfamily can function in a cooperative manner with SRF, and this is driven in part through the close juxta-positioning of their DNA binding sites, but also through direct protein-protein interactions [9]–[11]. Amongst the TCFs, ELK1 is the best studied and it is directly activated by phosphorylation in response to activation of the MAP kinase signalling cascades [reviewed in 7]–[8]. Mouse knockouts show minimal phenotypic changes [12], suggesting that there might be functional redundancy amongst members of this subfamily. This was recently demonstrated to be the case in the context of T-cell differentiation, where the loss of ELK1 caused only subtle effects and there was clear redundancy of function between ELK1 and ELK4 [13]. This redundancy of function was demonstrated at both the level of DNA binding and target gene regulation. Similarly, redundancy at the level of chromatin binding has been shown to occur in HeLa-S3 cells, where depletion of ELK1 led to decreased binding to chromatin, and a concomitant rise in the binding of ELK4 to the same regions in a subset of targets [14]. Thus, understanding the function of ELK1 and other TCFs is complicated by the compounding factors associated with functional redundancy in this transcription factor family. Recently however, a genome-wide RNAi screening study identified ELK1 as a critical factor in promoting cell survival in human breast-derived MCF10A cells [15]. MCF10A cells therefore provide a tractable system for dissecting ELK1 function.

Here, we have combined ChIP-seq and gene expression array analysis to interrogate the ELK1 target gene network in MCF10A cells. We demonstrate that ELK1 binds to its target regions in two distinct ways: uniquely or redundantly with other ETS proteins. The binding regions associated with these two types of interaction show different characteristics concerning the frequency and quality of the ELK1 binding sites and the association with the binding of heterotypic transcription factors. Unexpectedly, the two types of binding regions are associated with different modes of target gene regulation and moreover, this differential regulation also affects distinct functional categories of target genes. This is demonstrated for the unique ELK1 binding mode, where this factor controls cell migration, through acting in a positive manner to activate a set of functionally related target genes.

## Results

### Identification of an ELK1-regulated target gene network

**Figure 1:**
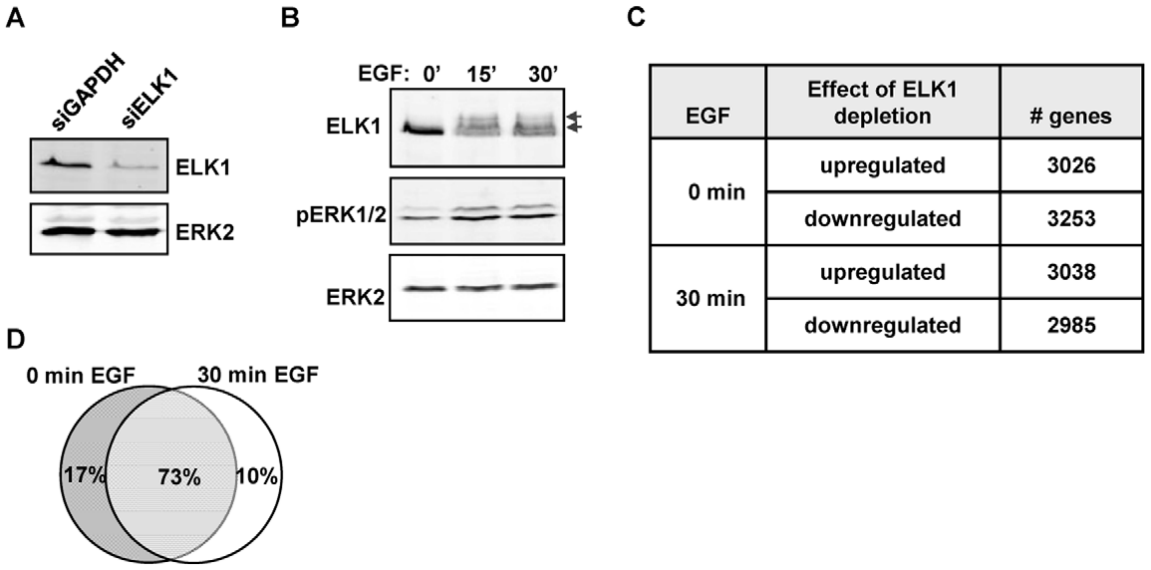
Depletion of ELK1 affects gene expression. Western blots of (A) ELK1 and ERK2 levels in MCF10A cells starved for EGF and treated with siGAPDH or siELK1 and (B) ELK1 and ERK2 phosphorylation levels in MCF10A cells following EGF treatment for the indicated times. Arrows indicate slower migrating phosphorylated ELK1 species. (C) Summary of microarray analysis of gene expression changes caused by ELK1 depletion in either EGF-starved or EGF-stimulated cells. The numbers of genes which become up- or down-regulated upon ELK1 depletion are indicated. (D) Overlap between genes which show a siELK1-induced change in mRNA levels at 0 min and 30 min of EGF stimulation.

Functional redundancy amongst members of multi-gene transcription factor families such as the ETS transcription factors is a potential problem when attempting to uncover the function of individual family members (reviewed in [1]). We therefore selected the human breast epithelia-derived MCF10A cells to dissect the role of ELK1 in controlling gene transcription, as ELK1 has been shown to be important for their survival and its deficiency cannot therefore be fully compensated for by other family members [15]. First, we depleted ELK1 levels in MCF10A cells and measured the effect this had on the transcriptome. The effect of ELK1 depletion was assessed in cells grown in the absence of EGF or in cells treated with EGF for 30 mins to activate ERK MAP kinase pathway signalling. ELK1 levels were efficiently reduced following treatment with siRNA (Figure 1A) and ERK activation and ELK1 phosphorylation were rapidly induced by EGF treatment (Figure 1B). Over 6000 genes consistently changed their expression (P-value<0.05; Q-value<0.1) following depletion of ELK1 either in the presence or absence of EGF, and roughly equal numbers of genes were up- and down-regulated in each case (Figure 1C; Table S1). In contrast, far fewer genes consistently changed their expression upon treatment with EGF (Figure S1), suggesting a more pleiotropic cellular response to ELK1 loss. Importantly, the majority of genes whose expression changed upon ELK1 depletion did so irrespective of the activity status of the ERK signalling pathway as only 10% of siELK1-sensitive genes were uniquely altered by ELK1 depletion in the presence on EGF, while 73% were changed both in the presence and absence of EGF (Figure 1D). This suggests a role for ELK1 in controlling gene expression which is largely independent from EGF signalling status.

**Figure 2:**
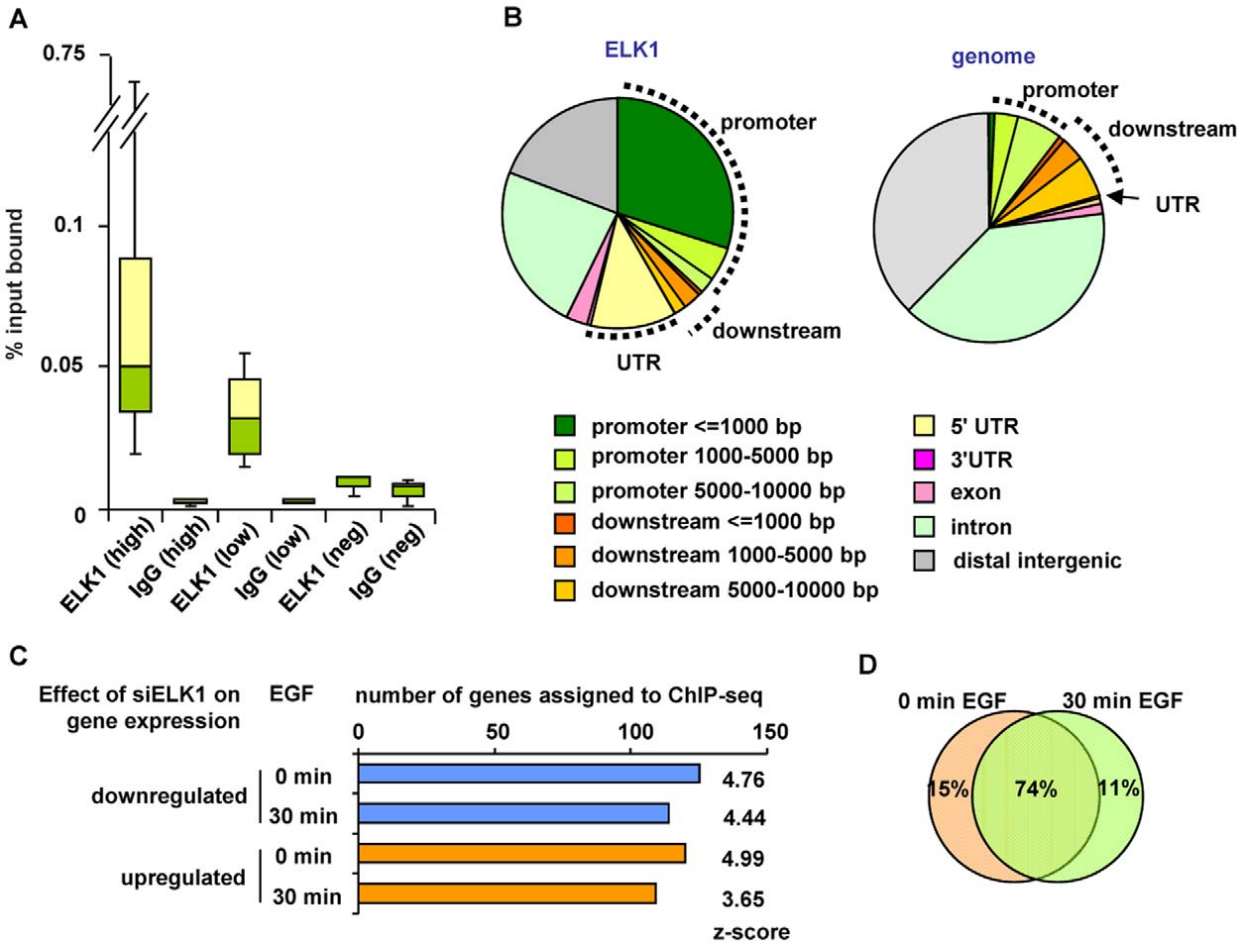
Identification of directly regulated ELK1 target genes. (A) Summary of ChIP-seq data validation (see Figure S2). Box plots show the median (horizontal lines) and the distribution of the enrichment levels, in qPCR validation of ELK1 or IgG ChIP samples. Binding regions were randomly selected from the high or low confidence ChIP-seq datasets. As a comparison, the values from regions which do not detectably bind ELK1 are shown (Neg). Data are from three biological repeats. (B) Genomic distribution of ELK1 ChIP-seq regions from the high confidence dataset (left) compared to a random distribution (right). Sectors corresponding to the promoter (up to 10 kb upstream from the TSS), the downstream region (up to 10 kb downstream from the TTS) and the UTRs are indicated. (C) Overlap between genes assigned to ELK1 ChIP-seq regions and genes which show a change in mRNA levels upon ELK1 depletion under the indicated conditions of EGF stimulation. Z-scores were obtained by comparing with an overlap of the ChIP-seq gene list with 10,000 random lists containing the same numbers of genes. (D) Overlap between numbers of genes which are associated with ELK1 binding events and show a siELK1-induced change in mRNA levels at 0 min and 30 min of EGF stimulation.

The large number of gene expression changes elicited in MCF10A cells by ELK1 depletion suggested that a sizeable proportion of the changes likely arise as an indirect consequence of ELK1 loss. Therefore we used ChIP-seq to establish the direct target genes for ELK1. These studies were performed in MCF10A cells grown in the absence of EGF, as ELK1 binding to chromatin is thought to occur irrespective of signalling conditions [16]. A total of 529 genomic binding regions for ELK1 were identified in two independent experiments (FDR<10), and denoted as a “high confidence” dataset (Table S2). By assigning binding regions to the nearest annotated TSS, 516 target genes were identified. A range of binding regions were validated by ChIP followed by qPCR and all the regions tested showed significant enrichment for ELK1 binding compared to a control non-specific antibody (Figure 2A; Figure S2). We also tested several regions from a lower confidence dataset where peaks were only identified in one of the two experiments and although these scored positive in qPCR-based assays, their relative enrichment levels were generally lower than observed for the high confidence data set (Figure 2A; Figure S2). Many of the regions in the high confidence dataset are also bound by ELK1 in a different human breast cell derived line, MDA-MB-231 cells (Figure S3A). Moreover, there is a large overlap of ELK1 binding regions with those identified in two previous studies using ChIP-chip (59%; [5]) and ChIP-seq (43%; [17]) in HeLa cells, with 106 (33%) regions in common between all three studies (Figure S4). This suggests that there is a core set of ELK1 binding regions common to several cell types but also a number of cell type-specific binding events. ELK1 binding regions in MCF10A cells are enriched in promoter -proximal regions with 36% within 10 kb upstream from the TSS of the nearest gene (Figure 2B). Functionally, the ELK1 binding regions were associated with genes grouped under a number of distinct gene ontology classifications by analysis using GREAT [18]. Prominent categories included a number of terms associated with the regulation of gene expression (e.g. “ribosomal subunit” and “mediator complex”) and with the actin cytoskeleton and cell adhesion (Figure S5). The association of ELK1 with genes encoding the core gene expression machinery was expected from previous results [5], but a role in potentially controlling genes involved in cytoskeletal-mediated events is a novel discovery.

Next, we assigned each ELK1 binding region to the nearest gene annotated in the RefSeq database, and compared this list with the ELK1-regulated genes revealed by expression microarray analysis. Over half of the genes associated with ELK1 binding regions (273 out of 516 genes) are also deregulated under at least one of the conditions we tested upon ELK1 depletion (245 were changed in the absence of EGF and 223 in the presence of EGF). Roughly equal numbers of ELK1 binding regions are associated with up- and downregulated genes, irrespective of EGF treatment (Figure 2C). Importantly, comparison of the ChIP-seq-derived ELK1 target genes with randomly selected gene sets demonstrated that the overlaps with the ELK1-regulated gene expression data are highly significant in all cases (z-scores ranging from 3.65 to 4.99). Further analysis of the results demonstrated that the majority of the 273 deregulated genes associated with ELK1 binding regions are the same irrespective of whether EGF was added to the cells or not (Figure 2D). However, amongst genes which are upregulated by EGF, there are 17 that are bound by ELK1, and of these, 13 show diminished expression following ELK1 depletion. This is in keeping with previous findings that ELK1 is associated with EGF/ERK pathway-mediated target gene activation but further illustrates that this appears to be a relatively minor role of ELK1 in this system. Functionally, the direct ELK1 target genes defined by this analysis retained categories identified in the entire ChIP-seq data set such as association with gene expression control and the actin cytoskeleton but in addition, a new category, apoptosis/cell death, was identified as being regulated by ELK1 (Figure S6A–S6C).

Together, these results identify a core set of direct ELK1 target genes, whose regulatory regions are bound by ELK1 and whose expression is perturbed upon depletion of ELK1. This set of genes likely represent directly regulated ELK1 target genes which we subsequently analysed further and are henceforth referred to as “ELK1 target genes”.

### ELK1 binding regions can be subdivided based on co-occurring regulatory features

**Figure 3:**
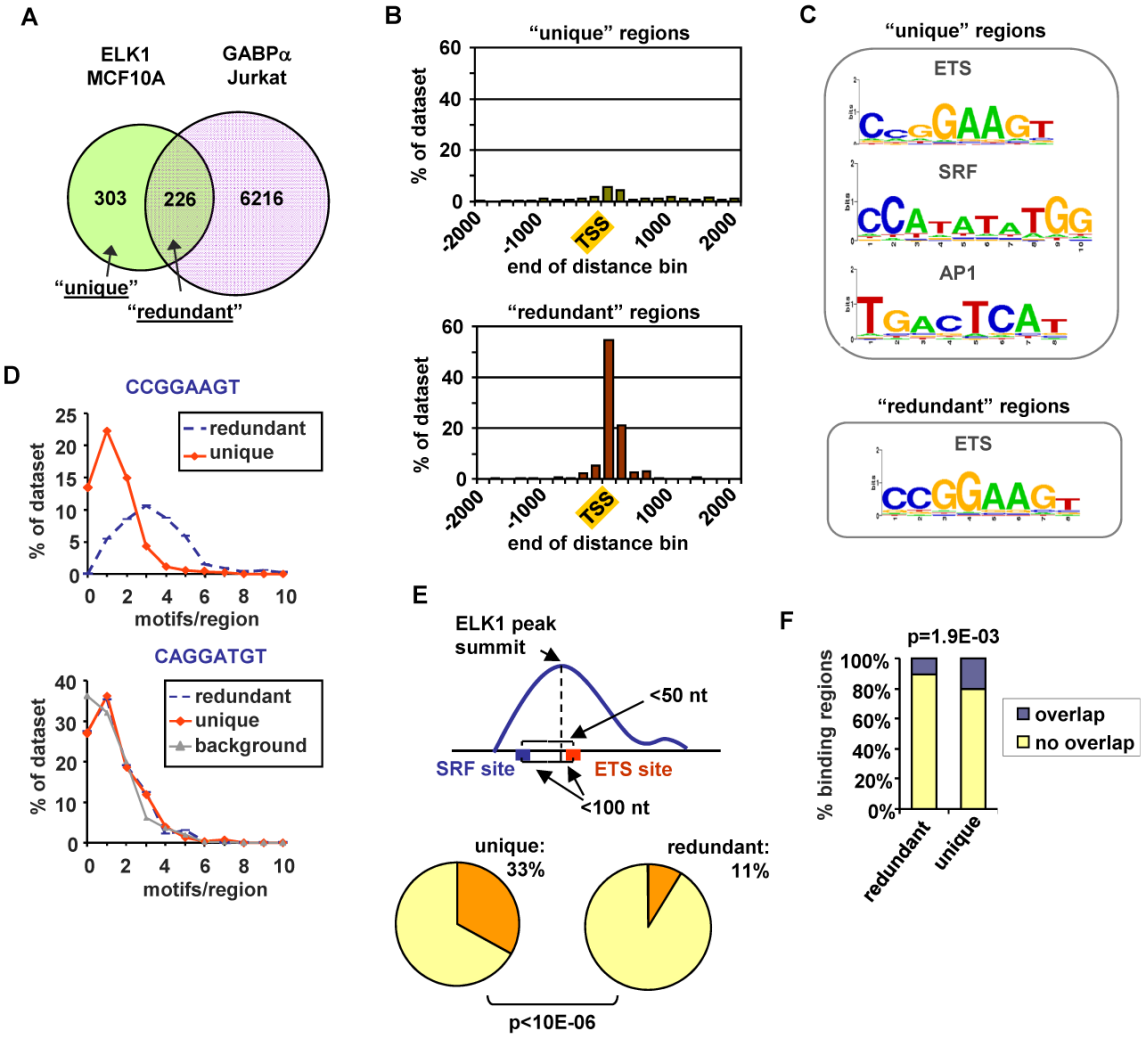
ELK1 binding region characterisation. (A) Overlap between regions bound by ELK1 (MCF10A cells) and GABPA (Jurkat cells; [17]). (B) Distribution of distances between peak summits and the nearest TSS for regions bound uniquely by ELK1 (“unique”-top) or redundantly with GABPA (“redundant” -bottom)(limited to within 2 kb up- or downstream). (C) TFBS logos obtained through STAMP-assisted visualisation of Weeder-derived position weight matrices of motifs overrepresented in “unique” (top) and “redundant” (bottom) ELK1-bound regions. Only the logos most closely resembling transcription factor binding sites present in the JASPAR database are shown. The second and third logos shown for the “unique” regions were identified by sequentially masking out the ETS and SRF binding motifs respectively. (D) Distributions of the number of ETS motifs found in the “unique” and “redundant” ELK1-bound regions. Occurrences of non-overlapping unique hexamers, corresponding to exact matches to derivatives of the octameric motifs shown above the graphs were counted. “Background” represents the distribution of an inverted ETS motif. (E) Occurrence of an ETS-SRF module in “unique” and “redundant” ELK1-bound regions. A module is defined as containing an ETS and SRF site within 100 bp of the ELK1 peak summit and a distance between the ETS and SRF motifs of less than 50 bp. (F) Overlap between the indicated categories of regions bound by ELK1 in MCF10A cells and regions bound by FOS in HeLa-S3 cells [18].

Previous ChIP-chip studies using promoter arrays demonstrated that ELK1 binding regions fall into three broad categories; regions that are also bound by SRF, regions that can also be bound by other ETS transcription factors and regions which are apparently uniquely bound by ELK1 independently from these other transcription factors [5], [14]. To establish whether we could detect similar overlaps with ELK1 binding regions on a genome-wide scale, we compared our ChIP-seq data with published ChIP-seq data for SRF and other ETS transcription factors. First, to address the potential for redundant binding of different ETS proteins, we compared the ELK1 ChIP-seq data with that of a divergent ETS factor GABPA [19]. A substantial overlap was observed between ELK1 and GABPA binding regions (43% of ELK1 binding regions) despite the differences in cell types analysed (Figure 3A). This overlap in binding suggests potential redundancy of binding site occupancy by these different ETS factors. We define the group of regions which can potentially be bound by both ELK1 and the divergent ETS protein GABPA as “ELK1 redundantly bound regions”, whereas those bound by ELK1 and not GABPA are termed “ELK1 uniquely bound regions”. To establish whether this subcategorisation held true when other ETS transcription factors were considered, we compared ChIP-seq data for nine other ETS transcription factors performed in a variety of cell lines with ELK1 binding regions which were classified as “unique” and “redundant”. With the exception of the highly related subfamily member ELK4, very little overlap was seen with binding regions in the “unique” subcategory but in 8/10 cases, there was significantly more overlap of these other ETS transcription factors with the “redundant” dataset (Figure S7B). Importantly, 67% of the “unique” ELK1 binding regions were not bound by any of the more divergent ETS proteins in any of these studies, supporting our subcategorisation of these regions. We also compared our ChIP-seq data with ChIP-seq data for SRF from Jurkat cells [19]. Again, a substantial overlap was seen between ELK1 and SRF binding regions despite the different cell types involved (28% of ELK1 binding regions; P<0.001) (Figure S7C). A comparison of ELK1 and SRF bound regions with the binding regions for GABPA permits further subcategorisation of binding events, and reveals a group of regions which are bound by ELK1 alone in the absence of potential redundant binding with GABPA or co-binding with SRF (Figure S7C).

In order to look for potential reasons for the non-identical transcription factor occupancy, we compared the regions bound uniquely by ELK1 (termed “unique”) and those which can also be bound by GABPA (termed “redundant”). First, we examined their location relative to the nearest TSS. The “unique” regions showed a broad distribution with only 26% being located within 2 kb of the TSS, whereas 93% of the “redundant” regions were located in this region and these were largely tightly centered around the TSS (Figure 3B). Next, we implemented a *de novo* search for over-represented DNA motifs within the binding regions. Three prominent motifs were identified in the “unique” dataset corresponding to ETS, SRF and AP1 binding sites, whereas the “redundant” dataset revealed only a motif corresponding to an ETS transcription factor binding site (Figure 3C). Similar results were obtained when using position weight matrices for all known human transcription factor binding motifs listed in the JASPAR and TRANSFAC databases to search for over-represented motifs in these two datasets (data not shown). The ETS motifs identified in the ELK1 binding peaks closely resembled the motifs identified in a recent high throughput *in vitro* binding site selection study [2] (Figure S8) and what was identified in a ChIP-chip study for ELK1 performed in HeLa cells [5], although differences in nucleotide preferences at several positions can be observed, generally being more stringent *in vitro*. Further inspection of the ELK1 binding regions demonstrated that the frequency of the three hexamers comprising the core CCGGAAGT binding motif was much higher in the “redundant” (763 sites in 226 regions) than in the “unique” dataset (403 sites in 303 regions). This also suggests that more than one binding event might be associated with “redundant” regions. Indeed, the number of motifs per region corresponding to hexameric derivatives of CCGGAAGT is significantly higher in the “redundantly” bound regions (commonly three sites per region) than in the “unique” regions (generally only one site per region) (Figure 3D). We investigated whether there were any particular spatial constraints among the multiple ETS sites found in the “redundant” regions but there was nothing obvious detected. In contrast to the differences seen when considering the core CCGGAAGT binding motif, the occurrence of hexameric derivatives of the variant ETS binding motif CAGGATGT is virtually identical in the two datasets (Figure 3D). However, some unique regions have only this relaxed variant, whereas all redundant regions have at least one “strong” consensus site. This suggests that the binding specificity is generally more divergent from the core consensus when unique binding of ELK1 is observed. Part of the reason for the relaxed binding specificity likely relates to the presence of co-occurring SRF binding motifs which are present in 54% of “unique” regions but only 27% of “redundant” regions. Indeed, a significantly greater number (P-value<1×10^−6^) of SRF motifs appear to form “modules” with adjacent ETS motifs in the “unique” dataset, where the two motifs co-occur within a 50 bp window (Figure 3E). It is possible that co-binding of factors to AP1 binding motifs also influences the binding specificity and functionality of ELK1, as the frequency of FOS binding to the same regions in a different ChIP-seq study [20] is significantly higher with the “unique” binding regions (Figure 3F). Interestingly, there are significantly more overlaps between ELK1 binding and FOS binding in regions associated with genes activated by ELK1 (27/141) rather than in genes where ELK1 acts in a negative manner (13/137) (P-value = 0.021), which is suggestive of a role for AP1 in enhancing the activity of ELK1. In keeping with a potential role for AP1 in modulating ELK1 function, the frequency of occurrence of AP1 binding motifs is also significantly higher in the “unique” binding regions (Figure S7D). Indeed, we can detect binding of the AP1 component FOS to several of the “unique” ELK1 binding regions (Figure S7F).

Together, these results therefore identify two major types of ELK1 binding regions which can be independently characterized by their locations, the types and numbers of DNA binding motifs they contain and the factors which can potentially co-occupy or compete for binding to these sites. Regions which are uniquely bound by ELK1 tend to contain fewer and/or weak ETS binding motifs and are often bound by other partner transcription factors such as SRF and AP1, whereas those redundantly bound by different ETS factors more often contain multiple strong ETS binding motifs, and there is little evidence for the occurrence of common co-binding transcription factors.

### Different ELK1 binding modes are associated with distinct categories of target genes

**Figure 4:**
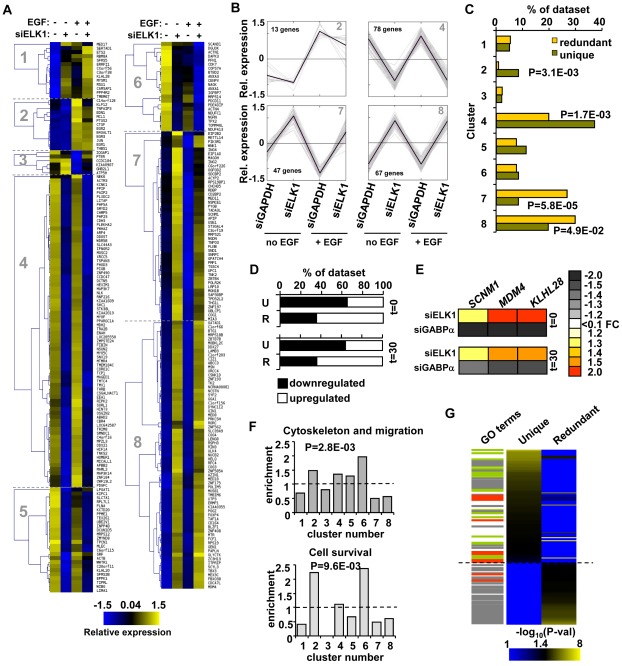
“Unique” and “redundant” ELK1-bound regions regulate distinct sets of target genes. (A) K-means clustering of expression levels of genes associated with ELK1-bound regions that show a significant response to siELK1 and/or EGF treatment. Individual clusters (1–8) are separated by dotted lines. (B) Summary profiles of the target genes in clusters 2, 4, 7 and 8. The data are presented as changes of individual (grey) and average (black) expression values of genes in each cluster under each of the four experimental conditions. For each gene, the mean of the signals across all four conditions was set as zero, and expression levels (z-transformed) presented are relative to this value. (C) Distribution of “unique” and “redundant” region-associated ELK1 target genes in the clusters identified in (A), with Chi square or Fisher Exact test-derived P-values. (D) Distribution of genes up- and down-regulated by ELK1 depletion in the “unique” (U) and “redundant” (R) ELK1 target gene datasets after 0 or 30 mins EGF stimulation. (E) Heatmap summary of changes in expression of three genes from the “redundant” ELK1 target dataset upon treatment with siELK1 (from microarrays) or siGABPA (by RT-PCR) (relative to a siGAPDH-transfected control), at 0 and 30 minutes of EGF stimulation. (F) Enrichment of the indicated GO categories in expression clusters identified in (A); P-values were obtained in a Chi-square test and refer to the cumulative enrichment of clusters which cross the dashed line *vs.* those that do not. (G) Heatmap of the distribution of GO terms identified for “unique” and “redundant” ELK1-associated genes. Each GO term is scored by −log_10_(P-value). Lines on the left mark terms related to gene expression (grey), actin/migration (green) and cell survival (red).

Having defined two different types of ELK1 binding regions, we next wished to investigate whether these regions are associated either with different gene regulatory mechanisms, and/or with controlling different cellular processes. First, we used unsupervised k-means cluster analysis to provide an unbiased way to identify groups of genes which responded differently to ELK1 depletion in the presence and absence of concurrent treatment with EGF. Here, we focussed only on the 273 direct target genes identified as being associated with regions bound by ELK1 in the ChIP-seq analysis. Data are depicted relative to average expression of each gene across all conditions to enable common regulatory patterns to be discerned. Eight gene expression clusters were identified which show distinct patterns of responses to these different treatments (Figure 4A, 4B; Figure S9; Table S3). We then examined whether the “unique” or “redundant” ELK1 target genes were associated with any particular cluster(s). Interestingly, we found a clear separation of target gene response associated with different ELK1 binding modes. Clusters 2 and 4 were significantly enriched for “unique” binding whereas clusters 7 and 8 were enriched for “redundant” binding (Figure 4C). Cluster 2 contains genes which are upregulated by EGF treatment but show an attenuated response upon ELK1 depletion, whereas genes in cluster 4 are largely unaffected by EGF but downregulated following ELK1 depletion (Figure 4A and 4B). In contrast, the genes in clusters 7 and 8 are largely non-responsive to EGF treatment but their expression is enhanced upon ELK1 depletion (Figure 4A and 4B). In total, over 75% (205) of the ELK1 target genes are found within the clusters which are strongly correlated with either “unique” or “redundant” binding. We analysed this further by comparing the response of genes bound either by ELK1 alone (“unique”) or by ELK1 and GABPA (“redundant”) (see Figure 3A) to ELK1 depletion. Here, a significant shift in the regulatory mode could clearly be seen, with “uniquely” bound genes being largely downregulated while “redundantly” bound genes were upregulated following ELK1 loss (Figure 4D). Mechanistically, one likely mode of regulation of the latter class of genes, is that ELK1 normally acts repressively at these genes and upon depletion is replaced by a different transcription factor such as GABPA which can potentially provide stronger gene activation. To provide data supporting this prediction, we compared cells depleted of either ELK1 or GABPA and examined the response of three genes predicted to be redundantly bound by ELK1 and GABPA. The expression of all three genes increased upon depletion of ELK1. However, the reciprocal occurred upon depletion of GABPA and decreased expression of all three genes was observed (Figure 4E). Thus, ELK1 and GABPA work antagonistically in controlling gene expression and this is particularly evident in clusters 7 and 8 for targets such as *SCNM1* and *MDM4*. Overall, these results indicate that the regulatory mode attributed to ELK1 strongly correlates with the type of ELK1 binding mode adopted on the target genes.

Next, we wanted to examine whether the type of binding and regulation adopted by ELK1 might also relate to functionally distinct outcomes in terms of the cohorts of genes regulated. We therefore examined whether any of the enriched GO term categories for the direct ELK1 target genes are preferentially associated with any of the different expression clusters. Importantly, we found that genes from particular functional categories are not evenly spread throughout the clusters (Figure 4F). Instead, genes forming the category “cytoskeleton and migration” are enriched in clusters 2, 4, 5 and 6, while genes within the “cell survival”-related terms are enriched in clusters 2 and 6. As one common defining feature of clusters 2, 4, 5 and 6 is downregulation following ELK1 depletion, these data suggest a positive role for ELK1 in driving transcription of genes in these functional categories. Therefore the gene expression clusters associated with particular ELK1 binding and regulatory modes are associated with distinct functional categories of genes. This suggests that the two types of ELK1 binding regions might regulate the expression of genes encoding proteins involved in distinct cellular processes and ultimately lead to distinct functional outcomes. To examine whether this is the case, we separated the direct ELK1 target gene dataset into “unique” and “redundant” groups according to the type of ELK1 binding mode, and screened these datasets for different functional categories of genes using DAVID [21]. We then ranked the resulting enriched GO terms according to statistical significance and saw a clear separation of these GO terms, according to association with either “unique” or “redundant” ELK1 binding with only minimal overlap (Figure 4G; Table S4). Genes “uniquely” bound by ELK1 were generally associated with terms related to the actin cytoskeleton (e.g. actin binding, focal adhesions etc.), whereas “redundantly” bound genes were generally associated with gene expression control (e.g. RNA processing, translation etc.). Some GO term categories related to gene expression were also associated with genes assigned to “uniquely” bound ELK1 regions but these strongly differed from the categories associated with genes assigned to “redundantly” bound regions, demonstrating a further separation of binding mode with respect to the types of genes regulated. Interestingly, terms related to cell survival were generally associated with genes assigned to “uniquely” bound ELK1 regions but also appeared in the “redundantly” bound dataset, albeit with lower significance.

These results therefore reveal an unexpected link between the mode of ELK1 binding to the regulatory region of a gene, the type of regulation of the associated genes and the functional classifications of the target genes controlled. In particular, genes associated with the cytoskeleton and migration, tend to be regulated in a positive manner by ELK1, and also are apparently bound specifically by ELK1 as they are not targets for potential redundant binding by alternative ETS transcription factors like GABPA.

### ELK1 controls cell migration

**Figure 5:**
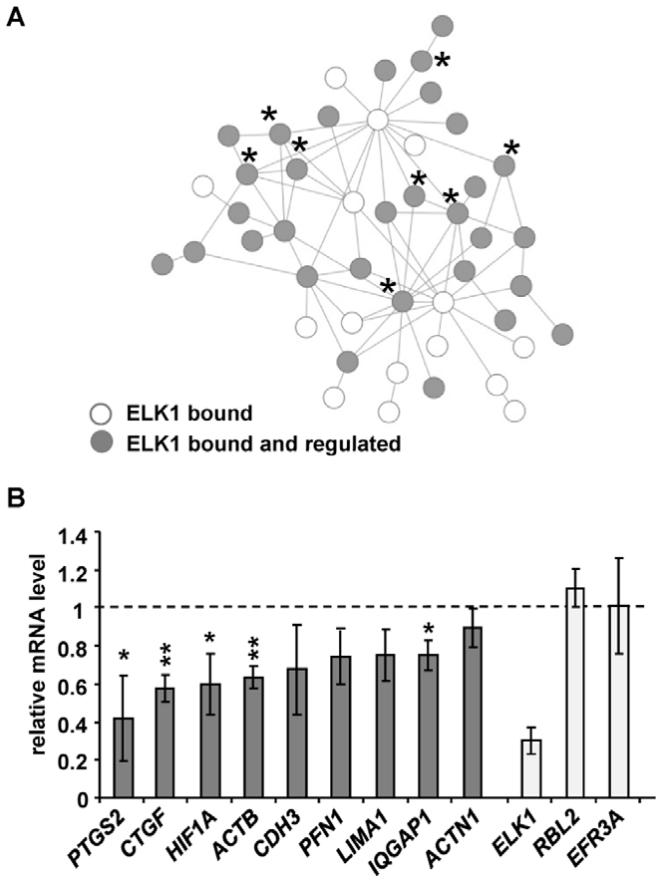
ELK1 controls a network of actin/migration-related genes. (A) Network formed by proteins encoded by actin cytoskeleton/migration-related genes associated with “unique” ELK1-bound regions (each protein denoted by a circle). Asterisks mark genes tested in (B) (*PFN1* is associated with a redundant region). Grey circles indicate that gene expression is changed upon ELK1 depletion. See Figure S11 for details of gene names. (B) mRNA levels of nine actin cytoskeleton- and migration-associated genes were measured by RT-qPCR in serum starved MCF10A cells transfected with siELK1 and normalised to an siGAPDH-transfected control; bars show average values from three biological repeats with standard deviations. Levels of *ELK1* mRNA indicate the efficiency of depletion; *RBL2*, *EFR3A* are negative controls which do not associate with ELK1. * P<0.05, ** P<0.01 (Student's paired t-test).

The above results indicate that a key role for ELK1 is mediated through its “unique” binding mode where it controls the activity of genes associated with the actin cytoskeleton and cell migration. To explore the functional significance of this association, we created networks of proteins encoded by “unique” and “redundant” ELK1 target genes using coexpression, textmining, knowledge and experimental data as proximity criteria to identify interconnectivities. These networks were screened for enrichment in GO terms centred on specific cellular functions. The genes associated with “migration/cytoskeleton” formed a prominent subnetwork within the “unique” dataset; more interestingly, a large number of the genes which form the core of this subnetwork were shown by microarray analysis to be misregulated following ELK1 depletion (Figure 5A and Figure S11). The role of ELK1 in controlling key nodes in this subnetwork was confirmed by qPCR. Nine different target genes were selected from the GO term category “migration/cytoskeleton”, whose regulatory regions are bound by ELK1. The majority of these showed decreases in expression upon ELK1 depletion from MCF10A cells in the absence and/or presence of EGF (Figure 5B, and Figure S10A). The majority of these decreases in ELK1 target gene expression were also observed in MDA-MB-231 cells (Figure S3B). Importantly, depletion of GABPA did not cause any significant changes in expression of these genes, in keeping with our designation of these genes as “unique” targets for ELK1 (Figure S10B). SRF has also been associated with controlling genes involved in the actin cytoskeleton and cell migration [22], [23]. We therefore also assessed the role of SRF in regulating the same group of genes. The expression of three genes was significantly reduced upon siRNA depletion (Figure S10C). Two of these, *CTGF* and *ACTB*, are also positively regulated by ELK1. Thus ELK1 and SRF appear to have both distinct and common targets amongst the “migration/cytoskeleton” gene network.

A second subnetwork of “cell survival” was also identified which is comprised of genes associated with the “unique” ELK1 dataset (Figure S12), but for the “gene expression” category, two different types of network could be observed (Figure S13A and S13B). In the latter case, again a subnetwork could be identified which is controlled through the “unique” binding of ELK1 and which features clusters of genes encoding signal-responsive transcription factors including immediate-early gene products and several nuclear hormone receptors. In contrast, an additional subnetwork was revealed which is apparently rather controlled through regions that can be redundantly bound by ELK1 and other ETS factors such as GABPA, and is mainly made up of genes encoding ribosomal subunits and mediator components. These findings support the association of different ELK1 binding modes with different functional categories of target genes, but also highlight the importance of ELK1 in controlling key nodes in the networks.

**Figure 6:**
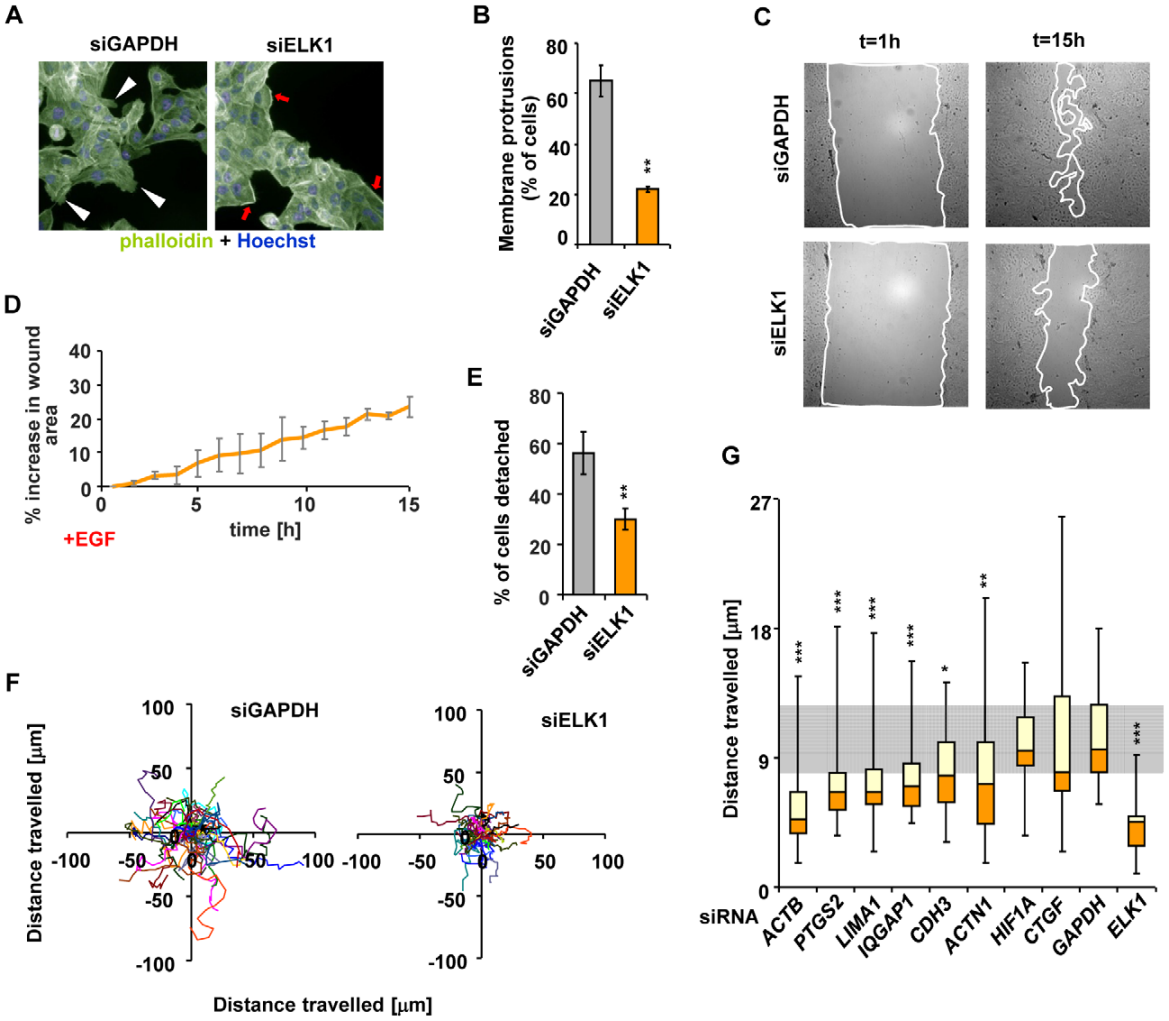
Depletion of ELK1 affects the cytoskeleton and the motility of MCF10A cells. (A) Control and ELK1-depleted MCF10A cells were starved for EGF for 48 h, subsequently stimulated with EGF for 24 hours and stained with phalloidin to visualise the actin cytoskeleton. Nuclei are stained with Hoechst (blue). Red arrows – subcortical actin, white arrowheads – membrane protrusions. (B) Quantification of the percentage of cells exhibiting membrane protrusions from siGAPDH- and siELK1-transfected cells. Bars show average values from three biological repeats with standard deviations; three fields were scored for each repeat. (C) Representative images of wounds created in monolayers of siGAPDH- or siELK1-transfected MCF10A cells, at t = 1 h and t = 15 h post-stimulation with EGF. Lines show borders of areas which were used for quantification. (D) Areas of wounds in monolayers of MCF10A cell treated as in (C) were measured at one-hour intervals of live imaging experiments; data are shown for the siELK1-treated population as a percentage increase of area compared to control siGAPDH treated cells. Graph shows average of three biological repeats with standard deviation. (E) Quantification of MCF10A cells which detached from clusters in the siGAPDH and siELK1-treated populations between t = 60 min and t = 460 min after EGF treatment. The experiment was performed in three biological repeats and in each case the fate of 20 cells was determined. (F) Migratory trajectories of MCF10A cells transfected with siGAPDH or siELK1, manually tracked between t = 1 h and t = 7 h of EGF stimulation. Each coloured line represents the path travelled by an individual cell. (G) Box plots of the distributions of trajectory lengths for MCF10A cells transfected with the indicated siRNA species, manually tracked between t = 1 h and t = 7 h after EGF stimulation. The experiment was performed in 3 biological repeats, in each case 10 cells were tracked. The grey shaded area corresponds to the second and third quartiles of the migratory range covered in control (GAPDH siRNA transfected) cells. Statistical significance was determined in Student's paired t-tests (B and F) or P-values were obtained from Kolomogorov-Smirnov tests (E and G). * P<0.05 ** P<0.01 *** P<0.005.

Our results predict that the loss of ELK1 should have profound consequences for the cellular phenotype due to the collapse of the networks involved with controlling the relevant cellular functions, especially in processes associated with the cytoskeleton and downstream effects such as cell migration. To test this, we first investigated the status of the cytoskeleton following ELK1 depletion in MCF10A cells. Following ELK1 depletion, cells became less spread and exhibited altered cytoskeletal characteristics, including a loss of membrane protrusions and enhanced levels of subcortical actin (Figure 6A and 6B). The loss of membrane protrusions suggested that the cells might lose their migratory properties. To test this, we used wound healing assays, and upon ELK1 depletion wound closure was greatly attenuated (Figure 6C and 6D). ELK1 depletion also led to reductions in cell numbers (Figure S14) which could at least in part contribute to the reductions in wound closure efficiency. We therefore used single cell tracking to examine the movement of individual cells associated with cell clusters. Fewer cells detached from these clusters in the siELK1-transfected population (Figure 6E) and those cells which did detach showed greatly impaired migration (Figure 6F). Many of the same phenotypic features could be observed upon ELK1 depletion in MDA-MB-231 cells (Figure S3C–S3E).

These results confirm that ELK1-mediated regulation of a target gene network associated with the actin cytoskeleton and cell migration correlates with the expected phenotypic consequences. To confirm that the key ELK1 target genes in this network play a role in controlling cytoskeletal-related activities such as cell migration, we individually depleted nine of these genes (Figure S15A) and analysed the resulting cellular phenotypes. Defects in the actin cytoskeleton (Figure S15B and S15C) and reduced wound healing (Figure S15D and S15E) were observed in several cases. Importantly, cell migration was defective in the majority of cases (Figure 6G and Figure S16), clearly demonstrating that the ELK1 target genes play a role in controlling this process.

## Discussion

Genome-wide studies are beginning to reveal the target genes for many transcription factors and one of the unanswered questions is how specific responses are generated by a particular transcription factor, especially in the presence of other proteins from the same family that can potentially bind the same sites. Here we have investigated the ETS family transcription factor ELK1 and made the unexpected discovery, that the types of binding mode exhibited by ELK1, correlate not only with the regulatory outcomes at the associated target genes but lead to the control of distinct networks of genes with defined cellular functions.

Our results clearly demonstrate that ELK1 target genes can be functionally classified according to their mode of ELK1 binding and this in turn controls their mode of regulation. Inspection of ELK1 binding regions demonstrates that these can be divided into at least three distinct categories based on the binding of other transcription factors; co-binding with a different transcription factor SRF, competition with GABPA (and potentially other ETS proteins) and binding in a distinct manner independently from these. To simplify our analysis, and concentrate on the potential interplay with other ETS transcription factor family members, we focused on two modes of ELK1 binding; regions which can be occupied by GABPA and ELK1 (“redundant” regions) or regions that can be occupied by ELK1 and apparently not GABPA (“unique regions”). While further analysis of the redundant and unique regions for binding by other ETS proteins from other ChIP-seq studies broadly supports this classification, it is important to emphasise that we cannot definitively conclude that the apparently “unique” regions cannot be bound by another ETS factor under different conditions or in another cell type. Nevertheless, this classification enabled us to ultimately define an important role for ELK1 in controlling cell behaviour where it acts through genes associated with the “unique” regions. These two types of binding regions showed distinct characteristics. “Redundant” regions are generally centered on the TSS and at the sequence motif level, contain multiple sites which closely match the generic consensus ETS protein binding motif CCGGAAGT. Moreover, there is little evidence for the occurrence of common co-binding transcription factors. In contrast, the “unique” regions are located in a wide distribution around the TSS and generally show a relaxed match to the ETS binding motif. Furthermore, these regions contain fewer ETS-like binding motifs than the “redundant” regions. The “unique” regions also show motif enrichment and binding of other classes of transcription factors; AP1 and SRF. The association between ELK1 and SRF has been well established previously (reviewed in [5], [24]) but the association with AP1 is a novel finding. This AP1 connection is related to early studies on the ELK1-regulated *FOS* promoter which suggested feedback inhibition by FOS [25]. Furthermore, recent genome-wide ChIP-seq studies demonstrate that the ETS proteins ERG, ETV1, and ETV4 exhibit strong co-binding with AP1 in prostate-derived cells whereas others (ETS1 and GABPA) do not [26]. Thus, co-association with AP1 appears to be a common mode of action for a subset of ETS transcription factors, including ELK1. Several other features of ELK1 binding have previously been reported from genome-wide binding analyses for other ETS transcription factors. For example, as observed for ELK1, ETS1 generally shows redundant occupancy of proximal promoters with GABPA but specific interactions with a coregulatory partner, RUNX1, generally take place in more distally located regions where ETS1 binds in a “unique” manner [4]. This study also revealed a putative link between “unique” binding of ETS1 and gene function with an association with T cell-specific genes being observed but this link remained functionally untested. Furthermore, the sequence characteristics of the core ETS1 binding sites differ between “unique” and “redundant” binding sites, as we observed in this study for ELK1. Thus, there are clear similarities in how ETS transcription factors generate specificity in their mechanism of action and biological functions. However, despite these underlying similarities, our study clearly demonstrates that ELK1 and ETS1 function in a different manner. Furthermore, our study was also able to make links to regulatory outcomes (see below) at different classes of target genes which have not yet been established for ETS1.

Importantly, the location of the ELK1 binding sites in proximal promoter (defined as +/−2 kb from the TSS) versus distal regions is not as effective at partitioning of the data. Indeed, the “unique” ELK1 binding sites in the proximal promoter regions share characteristics such as the distribution of binding motifs, over-represented functional categories of targets, and association of common regulatory events, with “unique” ELK1 binding sites located in the distal regions (data not shown). Thus, the mechanistic, regulatory, and functional connections that we observe are not due to binding site location and instead reflect whether ELK1 is likely working in a “unique” manner. However, when we partitioned the data according to a different definition of proximal binding (ie −7 kb to +2 kb from the TSS), we could readily detect SRF and AP1 motifs in the distal regions but ETS motifs were less obvious; the reciprocal was true in promoter-proximal regions (data not shown). Moreover, there were significantly more overlaps between distal ELK1 binding motifs with FOS ChIP-seq data [19] (58/229) than with promoter-proximal regions (20/300) (P-value<1×10^−6^). This might reflect a more combinatorial mode of action of ELK1 when it is associated with distal binding regions.

Once the two types of ELK1 binding mode had been established, we were then able to correlate binding with regulatory outcomes. “Uniquely” bound regions are generally associated with genes where ELK1 functions in an activating capacity, whereas the converse is true of “redundantly” bound regions. Direct ELK1 target genes which are upregulated by EGF treatment are generally associated with “unique” ELK1 binding regions, consistent with a role for ELK1 as an EGF-responsive transcriptional activator. Strikingly, the majority of ELK1 target genes were barely affected by EGF treatment, despite the fact that ELK1 plays a regulatory role at a large proportion of these. Thus although ELK1 is generally thought to function by acting as a direct recipient of ERK pathway signaling, this appears to be the exception rather than the rule when considering its broader cellular function (reviewed in [7]–[8], [24]). It should, however, be emphasised that even in the absence of EGF, basal ERK activation is present in MCF10A cells (Figure 1C) and thus we cannot rule out a role for constitutive low level signaling working through ELK1 to its target genes.

The lack of EGF response is particularly prominent among the “redundantly” bound ELK1 target genes. In these cases it appears likely that other ETS transcription factors such as GABPA can substitute for ELK1 binding, and the function of ELK1 is to compete for DNA occupancy with those ETS transcription factors. Indeed, depletion of ELK1 or GABPA has a reciprocal effect on the expression of “redundantly” bound target genes, suggesting that a dynamic equilibrium between these transcription factors exists in the cell to maintain target gene expression at the correct level. In this scenario, ELK1 would either be repressive in nature and GABPA activating, or alternatively, both might activate but with GABPA activating to a higher level than ELK1. Similar effects could occur amongst different family members as has been suggested previously, at least at the level of DNA binding [3]. Importantly, our data also suggest that while redundancy at the level of binding is observed, this binding might not translate into functional redundancy.

Given the different binding modes identified for ELK1 and their correlation with different regulatory outcomes for the associated target genes, we then wished to examine whether this translated into specific phenotypic responses. Unexpectedly, we uncovered a clear distinction between the type of ELK1 binding mode employed and the categories of target genes regulated. The “unique” ELK1 binding regions were closely associated with genes encoding proteins involved in the actin cytoskeleton and related processes, whereas the “redundantly” bound regions were generally associated with genes encoding proteins controlling aspects of gene expression. The latter observation suggests a general role for ETS proteins in maintaining the levels of genes encoding the gene expression machinery at the correct levels. A similar conclusion was reached based on overlapping binding of different ETS-proteins in a ChIP-chip study, where commonly bound target genes were often designated as “housekeeping” [3]. However, our results suggested a unique non-redundant function for ELK1 in controlling the expression of proteins associated with the actin cytoskeleton and we subsequently demonstrated that this was indeed the case. Upon ELK1 depletion, cytoskeletal defects were observed and these led to defects in cell migration. A previous study provided hints at such a connection with *MMP9* identified as an ELK1 target gene relevant to cell migration [27]. However, a direct link was not made between ELK1 and cell migration control and this gene was not identified as a direct target for ELK1 in our study.

Interestingly, SRF has also been associated with controlling the actin cytoskeleton and cell migration [22], [23] but the role of ELK1 as a potential partner protein in that process has not been addressed. Indeed, it is thought that the alternative SRF partner protein MAL/MRTF plays the major role in this process (reviewed in [28]). More recently, two different types of SRF binding sites have been shown to control cytoskeletal gene expression, where SRF binds either together or independently with the ETS transcription factor PU.1 and these binding events correlate with activation through ubiquitous promoter- and cell type-specific enhancer driven mechanisms, respectively [29]. Thus, different SRF binding modes are associated with the same biological process and type of regulation, while here we reveal that ELK1 binding modes dictate different downstream outcomes. Many of the target genes that are directly regulated by ELK1 differ from the SRF-regulated genes encoding proteins associated with the actin cytoskeleton. Indeed, of the 57 genes in the actin/migration network associated with ELK1 binding, only 14 were previously shown to be direct SRF targets in HeLa cells [5]. This suggests that these two transcription factors may control the expression of different components of the network rather than acting in a more general coordinated fashion. Indeed, we have shown that only a subset of the ELK1 target genes which rely on ELK1 for their expression, are also positively regulated by SRF. One of these, *ACTB*, encodes β-actin, and is a well established SRF target gene [e.g. 22], [ 23]. Importantly, we have confirmed the role of ELK1 in controlling cell migration in several human breast-derived epithelial cell lines, and it is possible that ELK1 may play a role in cellular metastasis in the context of breast cancer. The other major categories of ELK1-regulated target genes are associated with cell survival and apoptosis. However both “uniquely” and “redundantly” bound target genes appear to be involved. This association was expected due to the original observation that ELK1 is required for the survival of MCF10A cells [15].

In summary, this study has identified an intriguing link between different modes of transcription factor binding and the control of different functional categories of target genes. This permits a specific phenotypic response to be achieved depending on the intrinsic properties and the co-regulatory activities occurring at different transcription factor binding sites. In the case of ELK1, we uncover a specific role in controlling genes associated with the actin cytoskeleton and also determine that it has a second function in which it acts in a dynamic fashion with other ETS transcription factors to maintain the correct expression of components of the gene expression machinery. Future studies will attempt to uncover the mechanistic differences utilized by ELK1 in the different regulatory scenarios.

## Materials and Methods

### Tissue culture, RNA interference, and RT–PCR

MCF10A cells were maintained in DMEM/F12 containing 5% horse serum, 20 ng/ml EGF, 10 µg/ml insulin, 100 ng/ml cholera toxin and 0.5 µg/ml hydrocortisone. MDA-MB-231 cells were grown in DMEM containing 10% FBS.

For RNAi, MCF10A and MDA-MB-231 cells were plated out into a mixture of 83% growth medium, 17% OptiMEM (Invitrogen), 20 nM siRNA and 0.33% Lipofectamine2000 (Invitrogen) followed by replacement of the transfection mix with appropriate growth media after 12 hours. All siRNA constructs were ON-TARGETplus SMART pools from Dharmacon except for ELK1, where a custom-designed siRNA duplex corresponding to the sequence GGCAATGGCCACATCATCT was used, and for GABPA where additional duplexes were used from Santa-Cruz. For experiments involving the culture of siRNA-transfected cells longer than 64 hours, siRNA transfections were repeated at t = 48 hrs. For SRF knockdown followed by RT-PCR analysis, two repeats were done with an ON-TARGETplus SMART pool (Dharmacon) and one was performed with an alternative siRNA duplex (Santa Cruz).

Real time RT-PCR was carried out as described previously [30]. The primer pairs used for RT-PCR experiments are listed in Table S5.

### Expression microarray analysis

MCF10A cells were seeded into 6-well plates (520,000/well), transfected with siELK1 or siGAPDH (control) and maintained in media depleted of EGF for 48 hours. Cells were then stimulated with complete media containing EGF for 30 minutes (with non-stimulated populations used as control). Three biological replicates were performed for each condition. RNA was isolated using the RNeasy kit (QIAgene) according to the manufacturer's protocol and quantifiied with a Nanodrop ND-1000 spectrophotometer (Nanodrop Technologies). Sample labeling and hybridization to Affymetrix GeneChip Human Genome U133 Plus 2.0 arrays were performed according to manufacturer's instructions.

Upon collection of signal, technical quality control was performed with dChip (V2005) [31] using default settings. Background correction, quantile normalization, and gene expression analysis were performed using RMA in Bioconductor [32]. Principal component analysis (PCA) was performed with Partek Genomics Solution (version 6.5, Copyright 2010, Partek Inc., St. Charles, MO, USA). Differential expression analysis between samples was performed using Limma with the functions lmFit and eBayes [33]. A two-factor ANOVA model was used with batch pairing, since batch pairing was evident in the PCA analysis. Gene lists of differentially expressed genes were controlled for false discovery rate (FDR) errors using the method of QVALUE. All probesets with signals lower than or equal to the background level in the EGF treated control samples were discarded. For fold changes of signal between any two conditions, only probesets with Q values lower than 0.1 and P values lower than 0.05 were retained. For probesets complementary to multiple HUGO official gene symbols, only one gene name was retained. Probesets associated with gene names showing changes of signal in opposite directions were excluded from further analysis and if multiple probesets were associated with one official gene symbol, only the probeset exhibiting the largest change of expression was retained for further analysis. The microarray expression data have been submitted to array express (accession number E-MEXP-3407).

For clustering, signal intensities assigned to each probeset were log_10_-transformed and z-scores were calculated [34]. These transformed signal intensities were subsequently clustered in MultiExperimentViewer (MeV) [35] using the k-means algorithm with a preset of 8 clusters. These were subsequently organised using the hierarchical clustering algorithm.

### Wound healing, cell tracking assays, and immunofluorescent staining

Cells seeded in duplicate into 12-well plates were transfected with appropriate types of siRNA in media depleted of EGF (MCF10A cells) or FBS (MDA-MB-231 cells). After 48 hours wounds were created in each well using sterile pipette tips; wounds were scanned visually to ensure similar width. Cells were washed twice with 1× PBS and media containing either 20 ng/ml EGF (MCF10A cells) or 10% FBS (MDA-MB-231 cells) was added. For MDA-MB-231 cells, after 18 hours cells were stained with crystal violet. Four images were taken for each of the tested conditions, the area unoccupied by cells in each image was measured. Average values for each biological repeat were normalised to control (siGAPDH transfection). For MCF10A cells, upon stimulation plates were transferred to a chamber heated to 37°C and aerated with 5% CO_2_ and imaged for 15 hours. The area unoccupied by cells was measured at one hour intervals and normalised to values specific for the siGAPDH-transfected control. Alternatively, after 15 hours cells were fixed and stained with crystal violet. For single cell tracking, sparsely seeded cultured of MCF10A cells were treated identically as in wound healing assays (without the formation of wounds). All images were processed and analysed using ImageJ and Adobe Photoshop CS2.

Fixed cells were visualised using a Leica DMIL upright microscope coupled with a diagnostic instruments HRP045-NIK camera and a 20x/0.30 CPlan Ph1 – air objective. Images were acquired through the SPOT Basic software (Diagnostic Instruments) and processed in ImageJ and Adobe Photoshop CS2. Live cells were imaged in a heated, CO_2_-enriched chamber of a Leica DM IRE microscope equipped with motorised XYZ stages (Mauhauser) and a 20x/0.50 HC PlanFluotar (Ph2) objective. Images were acquired with a Coolsnap HQ CCD camera (Photometrics) through the Image Pro 6.3 software (Media Cybernetics LtD) and later processed using ImageJ.

For immunofluoresence experiments, cells were fixed with 3.7% paraformaldehyde in 1×PBS, permeabilised with 0.01% Triton X-100 in 1×PBS, washed twice with 1× PBS, stained for 20 min with a solution of AlexaFluor488 phalloidin (Invitrogen), then washed three times with PBS. For detecting DNA, cells were co-stained with a 1 µg/ml solution of the Hoechst dye. Cells were imaged using Olympus BX51 upright microscopes equipped with 20x/0.50 UPlanFln – air objectives and captured with a Coolsnap HQ camera through MetaVue Software (Molecular Devices).

### Western blot analysis

Western blotting was carried out with the primary antibodies; ELK1 (Epitomics, #1277-1), cFOS (SantaCruz, sc7202), ERK2 (SantaCruz, sc154), and phospho-ERK (Cell Signalling, 9106S). The proteins were detected either by chemiluminescence with SuperSignal West Dura Substrate (Pierce) and visualised with a Fluor-S MultiImager (Bio-Rad) or for infrared dye-conjugated antibodies, signal was collected with a Li-cor Odyssey Infrared Imager.

### Chromatin immunoprecipitation (ChIP) assays

ChIP assays using control IgG (Millipore) or antibodies specific to ELK1 (Epitomics) or cFOS (Santa Cruz) were performed essentially as described previously (O'Donnell et al. 2008) using 3×10^6^ MCF10A cells for a standard ChIP and 5.4×10^7^ cells for a ChIP-seq experiment, grown in media depleted for EGF for 48 hrs for ELK1 ChIP or following stimulation with EGF re-addition for 2 hours for FOS. Bound promoters were detected by quantitative PCR using primers listed in Table S5. Quantitative PCR was performed at least in duplicate, from at least three independent experiments, and analysed as described previously [5].

### ChIP–seq assays

Samples for ChIP-seq were prepared as described above and libraries were generated and sequencing was performed on a Life Technologies SOLiD 3.5 System (Applied Biosystems) according to the manufacturer's protocols. Two repeats were performed.

SOLiD (Applied Biosystems) *.csfasta sequencer reads were truncated to 32 nt using a perl script: *SOLiD_preprocess_filter_v1.pl*, available at http://hts.rutgers.edu/filter/. Reads were then aligned to build 18 of human genome from March 2006 (hereafter referred to as hg18) using Corona-Lite 4.2.2 (Life Technologies) and allowing from 0 to 3 mismatches. Aligned reads were used for identification of peaks by MACS v. 1.3.7.1 [36]. For each of the two repeats, the ELK1 signal was compared sequentially to the IgG and input signals and the overlaps of the resulting sets of regions were carried out using Galaxy [37], [38]. The resulting sets of ELK1-specific peaks for each repeat experiment were once more overlapped and a high confidence dataset was defined as regions identified in repeat 2 that overlapped those identified in repeat 1. Regions identified only in repeat 2 are considered to be low-confidence targets. In both cases, only regions characterised by a False Discovery Rate (FDR) lower than 10% were retained. The ChIP-seq data have been submitted to array express (accession number E-MTAB-830).

For association of ELK1 binding regions with potential regulated genes, each ChIP-seq region was assigned to its nearest gene annotated in the refGene table (release 41, May 2010) of the RefSeq Genes track, downloaded from the UCSC Table Browser. This was based on the position of summit of each peak (identified by MACS) and the position of the TSS of the relevant RefSeq gene. Overlaps between ChIP-seq-associated genes and microarray-generated gene expression data were determined using an online tool (http://jura.wi.mit.edu/bioc/tools/compare.php).

### Bioinformatics analysis

The genomic distribution of peaks was determined using CEAS [39]. *De novo* motif discovery was carried out using Weeder v. 1.4.2 [40]. The resulting top scoring position weight matrices (PWMs) were parsed against known transcription factor PWMs from the JASPAR 2010 database [41] using STAMP [42].

Word-based motif searches were performed using two PERL scripts: *CountRegexGFF_IUPAC_1input_nosummary.pl* (identifies number of occurrences of a particular IUPAC string on one or both strands of one or more sequences provided in FASTA format) and *CountRegexGFF_IUPAC_1input_simple_output.pl* (returns positions of all occurrences of a given IUPAC string within given FASTA sequences).

For constructing networks, lists of gene names assigned to ChIP-seq regions were uploaded into STRING [43]. The resulting networks were saved as *.txt files and then uploaded into Cytoscape (v. 2.7.0) with cumulative scores of coexpression, textmining, knowledge and experimental data as proximity criteria. yFiles→organic network layouts were applied and the positioning and graphic representation of nodes were adjusted manually for increased clarity.

For functional profiling of ELK1 binding regions identified by ChIP-seq, Gene Ontology was performed using GREAT v. 1.2.6 [18] with the default (basal plus extension) gene regulatory definition option. Here, each ChIP-seq region is assigned to all genes whose domains it overlaps. Significance was determined by FDR<0.05, and terms significant by both the region-based binomial test and gene name-based hypergeometric test are reported. Gene Ontology analysis of lists of gene names was performed using DAVID v. 6.7 [21] and terms with P-values<0.05 were retained. In each case, Official Gene Symbol was used. Gene Ontology of networks was carried out using the Cytoscape plugin BiNGO (v. 2.42) [44] with default settings.

### Statistical analysis

Statistical analysis for qRT-PCR studies and ChIP assays were performed using paired, 2-tailed Student's t test. The error bars in all graphs represent standard deviation. Fisher's Exact tests and Chi-square tests were used to compare two different distributions of pairs of datasets with respect to a particular condition. Z-score analysis of overlaps between two gene name lists was performed using a PERL script using 10000 background lists of Official Gene Names of size of one of the comparator datasets. Average overlap with standard deviation was recorded and z-score was calculated as the ratio of difference between actual overlap and this average overlap over the standard deviation. Kolomogorov-Smirnov tests were used to determine the statistical significance of differences in shape and range between two distributions. The Genomic HyperBrowser was used to determine the statistical significance of overlaps of sets DNA regions with the following settings: regions of dataset 1 fixed, region sizes and number of dataset 2 fixed, tested hypothesis: overlaps more than expected, overlap tested for the whole genome, with each chromosome treated as a separate bin (maximum possible bin size).

### Data release

The ChIP-seq and microarray expression data have been submitted to array express (accession numbers E-MTAB-830 and E-MEXP-3407) and the data will be released upon publication.

## Supporting Information

Figure S1 Summary of microarray analysis of gene expression changes in either siGAPDH or siELK1-treated cells upon EGF stimulation. The numbers of genes which become up- or down-regulated upon EGF stimulation are indicated.
(PDF)

Figure S2 Validation of ChIP-seq datasets. qPCR validation of a range of ELK1 binding regions identified by ChIP-seq. Binding regions were ordered by descending tag count and candidates were selected by taking binding regions approximately every fiftieth and every one-hundredth position in the lists from the high and lower confidence ELK1 ChIP-seq datasets. ChIP-seq peak profiles for the ELK1 (blue vertical line) and IgG (yellow vertical line) samples are shown on the left. Bars in profile windows indicate 500 nt; RefSeq annotated genes are shown (if present) and the TSS is indicated by an arrow. The signal range (y-axis) is 0 to 100 for all regions apart from region 2 in the high confidence dataset (*WNK1*), where the maximum reaches 225. Specific binding of ELK1 to each region was validated in ChIP-qPCR experiments carried out in three biological repeats (shown on the right of the corresponding peak profiles); bars show the average percentages of input precipitated with the ELK1 antibody (blue bars) or non-specific IgG (yellow bars) with standard deviations. Titles of the graphs indicate the position of each region on a list ordered with descending tag count of ELK1 signal (prefix “L” denotes position on lower confidence list) and the name of the nearest annotated RefSeq gene. Binding regions from the lower confidence dataset are boxed. *ABLIM2*, *CPLX1* and intron 3 of the SRF gene (*SRFint3*) are negative controls and *EGR2* is a positive control. Data are summarized in Figure 2A.
(PDF)

Figure S3 Role of ELK1 in MDA-MB-231 cells. (A) ChIP-qPCR experiments were carried out in MDA-MB-231 cells for regions bound by ELK1 in MCF10A cells (see Figure 2A, and Figure S2; only the high confidence dataset was tested). Bars show the average percentages of input precipitated with the ELK1 antibody (black bars) or non-specific IgG (grey bars) with standard deviations. Numbers above bars show fold enrichment of ELK1 signal over IgG. *EGR2* – positive control, *ABLIM2*, *CPLX1*, *SRFint3* – negative controls. (B) The effect of depletion of ELK1 on mRNA levels of the indicated actin cytoskeleton- and migration-associated genes. RT-qPCR analysis of the mRNA levels of the indicated genes was carried out in serum starved MDA-MB-231 cells transfected with siELK1, with normalisation to an siGAPDH-transfected control; bars show average values from three biological repeats with standard deviations. Levels of *ELK1* mRNA indicate the efficiency of depletion and *RBL2* and *EFR3A* are control genes not associated with ELK1 Binding regions; * P<0.05, ** P<0.01 (Student's paired t-test). (C) Representative images of wounds created in monolayers of siGAPDH- or siELK1-transfected MDA-MB-231 cells, 18 hours post-stimulation with media containing 10% FBS. Lines show borders of areas which were used for quantification. (D) Areas of wounds in MDA-MB-231 cells treated as in (C) were measured in duplicates and normalised to control (siGAPDH). Bars show the average of three biological repeats with standard deviation. P-value was obtained from a two-tailed paired Student's t-test. (E) Numbers of MDA-MB-231 cells in a siELK1-transfected population were counted at 48 and 96 hours post-initial transfection and normalised to control (siGAPDH). Bars show average values of three biological repeats (performed in duplicates) with standard deviations. * P<0.05 (Student's paired t-test).
(PDF)

Figure S4 Overlaps between ELK1 binding regions in MCF10A cells and HeLa cells. (A) Overlap between regions identified by ChIP-seq as bound by ELK1 in MCF10A cells, and by ChIP-chip as bound by ELK1 in HeLa cells [5]. (B) Overlap between regions identified by ChIP-seq as bound by ELK1 in MCF10A cells, and by ChIP-seq as bound by ELK1 in HeLa cells [17]. The ChIP-seq data from MCF10A cells is partitioned into promoter proximal (−10 kb to +2.5 kb from the TSS) and distal binding regions to enable comparison with the ChIP-chip data which only samples this region of the genome.
(PDF)

Figure S5 Gene Ontology analysis of regions bound by ELK1. GREAT Gene Ontology analysis was carried out for all regions from the high confidence dataset using default settings. Significantly enriched categories are shown. Categories associated with the actin cytoskeleton and migration are indicated by arrows.
(PDF)

Figure S6 Depletion of ELK1 changes the expression of distinct functional categories of genes. (A) DAVID functional clustering analysis was carried out for genes assigned to ELK1 ChIP-seq regions which show a significant change in expression levels in MCF10A cells depleted of ELK1 (as compared to an siGAPDH-transfected control), at either time point of EGF stimulation. Functional assignments of the top ten clusters are shown. GO terms were classified as gene expression-related (grey), actin cytoskeleton/migration-related (green) and cell survival related (orange). (B) DAVID functional clustering results for genes upregulated upon ELK1 depletion; the top five clusters are shown. (C) As in (B), but for downregulated genes.
(PDF)

Figure S7 Overlaps between ELK1 binding regions and binding of other transcription factors; FOS/AP1 binding associates with “unique” ELK1-bound regions. (A) Overlap between regions identified by ChIP-seq as bound by ELK1 (MCF10A cells) and ELK4 (HeLa cells) [17]. (B) Overlap between regions identified by ChIP-seq as bound by ELK1 (MCF10A cells) and the indicated ETS transcription factors [2], [17], [24]. RPWE1 are normal prostate, Jurkat are T cell lymphoma, VCaP are prostate cancer, HL60 are leukaemia and HeLa are cervical cancer cells. The data are compared to the 303 “unique” and 226 “redundant” ELK1 binding regions, and the % overlap is presented (relative to the number of ELK1 regions). P-values are calculated in Chi square tests and significance values assigned to datasets which show a preferential differential enrichment with the “redundant” ELK1 binding regions. (C) Overlap between regions identified by ChIP-seq as bound by ELK1 (MCF10A cells), SRF (Jurkat cells; [19]) and GABPA (Jurkat cells; [19]). (D) Occurrence of FOS/AP1 motifs corresponding to the TGANTCA consensus (purple quadrants) in “unique” and “redundant” ELK1-bound regions. P-values were calculated using a Chi square test. (E) Western blot showing expression levels of FOS in MCF10A cells at indicated times after EGF addition. (F) Binding of FOS to “unique” (*WNK1*, *ITGAV*, *PAPLN*) and “redundant” (*CAP1*) regions was determined in MCF10A cells two hours post-EGF stimulation in ChIP-qPCR assays. Numbers above bars indicate fold enrichment of FOS signal over IgG. *KLF9* – positive control.
(PDF)

Figure S8 Comparison of ELK1 binding motifs found *in vivo* and *in vitro*. TFBS logos obtained through STAMP-assisted visualisation of Weeder-derived position weight matrices of motifs overrepresented in “unique” and “redundant” ELK1-bound regions from the ChIP-seq experiment in MCF10A cells (see Figure 3C). For comparison, the logo for the ELK1 binding site obtained from an *in vitro* site-selection study [2] is shown at the bottom.
(PDF)

Figure S9 “Unique” and “redundant” ELK1-bound regions regulate distinct sets of target genes. Summary profiles of the target genes in clusters 1, 3, 5 and 6 (see Figure 4A). The data are presented as changes of the individual (grey) and average (black) expression values of genes in each cluster under each of the four experimental conditions. For each gene, the mean of the signals across all four conditions was set as zero, and expression levels (z-transformed) are presented relative to this value.
(PDF)

Figure S10 The role of ELK1, GABPA and SRF in regulating “unique” ELK1 target genes. (A–C) mRNA levels of nine actin cytoskeleton- and migration-associated genes were measured by RT-qPCR from MCF10A cells grown in the absence of EGF (B and C) or additionally treated with EGF for 30 mins (A) and transfected with siRNAs against ELK1 (A), GABPA (B) or SRF (C); these were then normalised to an siGAPDH-transfected control (taken as 1). Bars show average values from two to three biological repeats with standard deviations. Levels of *ELK1*, *GABPA* and *SRF* mRNAs indicate the efficiency of depletion; *RBL2*, *EFR3A* are negative controls which do not associate with ELK1. Significantly altered expression is depicted with orange bars; * P<0.05, ** P<0.01 (Student's paired t-test).
(PDF)

Figure S11 ELK1 controls a network of actin/migration-related genes. Network formed by proteins encoded by actin cytoskeleton/migration-related genes associated with “unique” ELK1-bound regions (each protein denoted by a circle). Asterisks mark genes tested in Figure S10 and Figure 5A (*PFN1* is associated with a redundant region). Pink circles indicate that gene expression is changed upon ELK1 depletion. Circles to the right of the figure show ELK1 target genes attributed to these functional terms but which do not have catalogued connections to the other genes in the network.
(PDF)

Figure S12 ELK1 controls a network of survival-related genes. Networks formed by proteins encoded by survival-related genes associated with “unique” or “redundant” ELK1-bound regions (each protein denoted by a circle). Asterisks mark genes tested in Figure 5A and Figure S10, Pink and green circles indicate that the genes are associated with “unique” ELK1 binding regions and their gene expression is changed or unchanged respectively upon ELK1 depletion. Yellow and blue circles indicate that the genes are associated with “redundant” ELK1 binding regions and their gene expression is changed or unchanged respectively upon ELK1 depletion. Circles to the right of the figure show ELK1 target genes attributed to these functional terms but which do not have catalogued connections to the other genes in the network.
(PDF)

Figure S13 ELK1 controls a network of gene expression-related genes. Networks formed by proteins encoded by gene expression-related genes associated with “unique” (A) and “redundant” (B) ELK1-bound regions. Asterisks mark genes tested in Figure S10, Figure 5A. Pink and yellow circles indicate that the genes are associated with “unique” and “redundant” ELK1 binding regions and their gene expression is changed upon ELK1 depletion whereas green and blue circles indicate that the genes are associated with “unique” and “redundant” ELK1 binding regions and their gene expression is unchanged upon ELK1 depletion. Circles to the right of the figure show ELK1 target genes attributed to these functional terms but which do not have catalogued connections to the other genes in the network. Subclusters of proteins that correspond to distinct categories of regulators are boxed.
(PDF)

Figure S14 Depletion of ELK1 impairs MCF10A cell survival. The numbers of MCF10A cells were determined 48 h and 96 h post-initial treatement with ELK1 siRNA and normalised to siGAPDH-transfected control. Cells were stimulated with EGF at t = 48 h (together with the second transfection). The experiment was performed in duplicate; average values and standard deviations from three biological repeats are shown. Significance was determined in a Student's paired t-test, * P<0.05.
(PDF)

Figure S15 ELK1-regulated genes control the actin cytoskeleton and motility of MCF10A cells. (A) Depletion levels of each of the indicated mRNA species following siRNA treatment were determined in RT-qPCR reactions. Bars show average values from three biological repeats with standard deviations, and are shown relative to cells treated with siGAPDH (taken as 1). (B) Representative images of MCF10A cells transfected with the indicated siRNA species, starved for EGF for 48 h and subsequently stimulated with EGF for 24 h and stained with phalloidin (green) and the Hoechst dye (blue). (C) The percentage of cells exhibiting membrane protrusions was calculated for each of the indicated siRNA transfections, as well as for siGAPDH (negative) and siELK1 (positive) – transfected controls. Bars show average values from three biological repeats with standard deviations; three fields were scored for each repeat. (D) Representative images of wounds created in monolayers of MCF10A cells transfected with the indicated siRNAs, are shown 15 hours post-stimulation with EGF (cells were stained with crystal violet). (E) Areas of wounds in MCF10A cells treated as in (D) were measured in duplicates and normalised to control (siGAPDH). Bars show average values of three biological repeats with standard deviations. P-values were calculated in Student's paired t-tests, * P<0.05
(PDF)

Figure S16 Depletion of ELK1 target genes impairs MCF10A cell migration. Migratory trajectories of MCF10A cells transfected with the indicated mRNA species, manually tracked between t = 1 h and t = 7 h of EGF stimulation. Each coloured line represents the path travelled by an individual cell.
(PDF)

Table S1 Microarray analysis of gene expression changes upon EGF treatment and/or depletion of ELK1 in MCF10A cells. Data are shown for (A) all the genes which show significant changes in expression upon EGF treatment and/or depletion of ELK1 and (B) genes which show significant changes in expression upon EGF treatment and/or depletion of ELK1 and are also associated with ELK1 binding regions. Only probesets with Q values lower than 0.1 and P values lower than 0.05 were retained, and only one probeset per gene was considered, see Materials and Methods for details.
(XLSX)

Table S2 ChIP-seq analysis of ELK1 binding regions in MCF10A cells. Data are shown for (A) high confidence binding regions which have a FDR<10% and appear in two independent experiments and (B) lower confidence binding regions which have a FDR<10% but appear in only one experiment. Genomic coordinates, summits, tag numbers and significance values are shown for each region from the experiment (repeat 2) which gave peaks with more stringent P-values.
(XLSX)

Table S3 Z-transformed mRNA expression data for clustering analysis. The mRNA expression changes for genes associated with ELK1-bound regions that show a significant response to siELK1 and/or EGF treatment are shown as z-transformed data for each of four different experimental conditions, i.e. plus/minus siELK1 treatment, plus/minus EGF treatment for 30 mins. This data was used for generating the k-means clustering of expression levels shown in Figure 4A.
(XLSX)

Table S4 “Unique” and “redundant” ELK1-bound genes associate with distinct sets of GO terms. DAVID Gene Ontology was performed on lists of genes associated with “unique” or “redundant” ELK1-bound regions that changed expression upon depletion of ELK1. Lists were sorted with ascending P-values of individual terms. These data are shown as a heatmap in Figure 4G.
(XLSX)

Table S5 List of PCR primers used in (A) ChIP and (B) RT-PCR experiments. Nucleotide sequences are given in both cases, and the chromosomal locations of the primers used for ChIP (according to the hg18 genome assembly).
(PDF)
