# Supplementary material for: ELK1 Uses Different DNA Binding Modes to Regulate Functionally Distinct Classes of Target Genes
Source: PLoS Genet. 2012 May 10;8(5):e1002694. doi: 10.1371/journal.pgen.1002694 (PMC3349735; doi:10.1371/journal.pgen.1002694)
Supplement: Figure S1 — Summary of microarray analysis of gene expression changes in either siGAPDH or siELK1-treated cells upon EGF stimulation. The numbers of genes which become up- or down-regulated upon EGF stimulation are indicated. (PDF) [file pgen.1002694.s001.pdf]

| siRNA | Effect | # genes |
|---|---|---|
| GAPDH | upregulated | 137 |
| | downregulated | 69 |
| ELK1 | upregulated | 92 |
| | downregulated | 0 |

# Supplementary Figure S1
