# Supplementary figures and images for: ELK1 Uses Different DNA Binding Modes to Regulate Functionally Distinct Classes of Target Genes
Source: PLoS Genet. 2012 May 10;8(5):e1002694. doi: 10.1371/journal.pgen.1002694 (PMC3349735; doi:10.1371/journal.pgen.1002694)

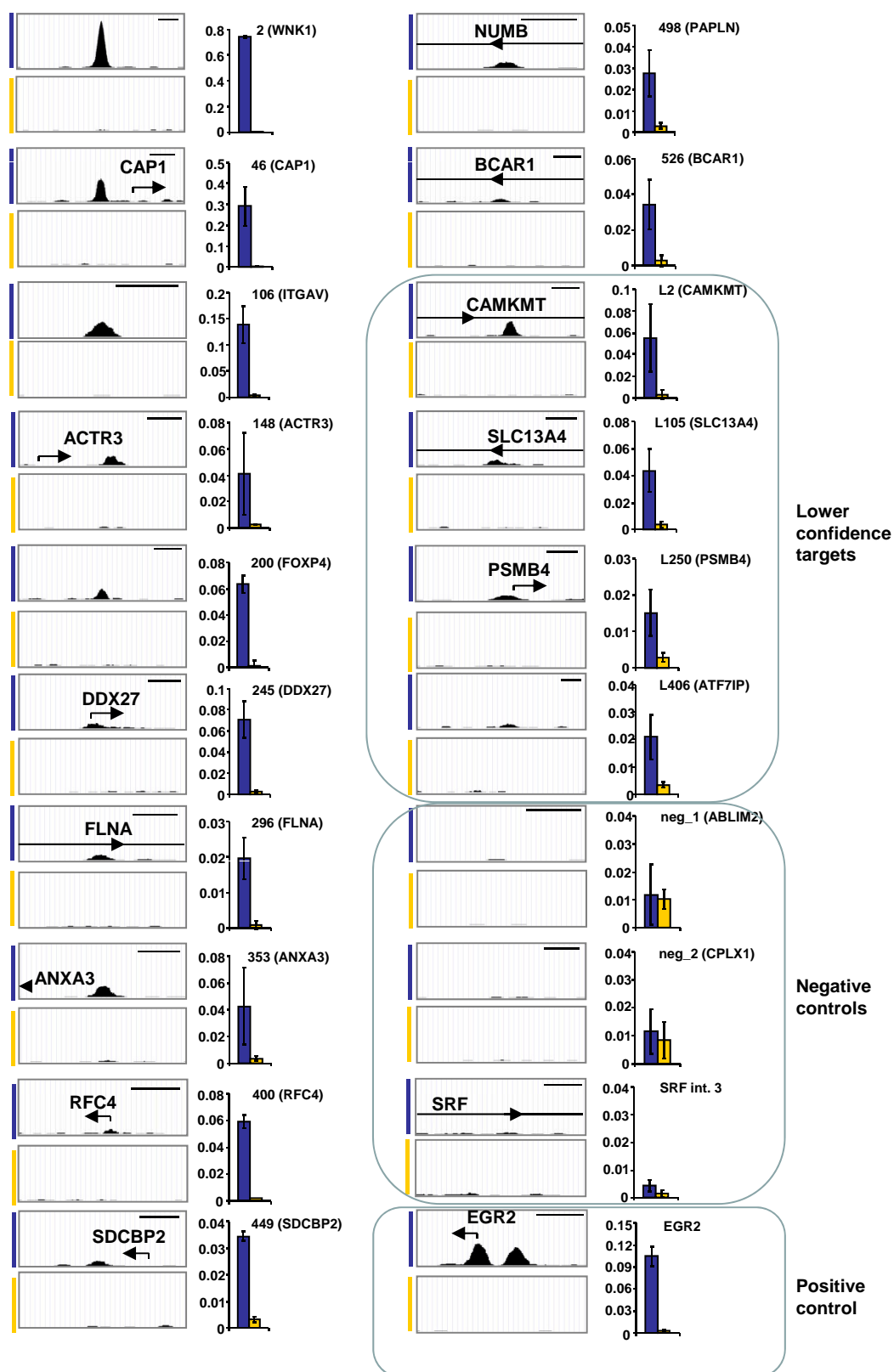

Supplementary Figure S2

Supplement: Figure S2 — Validation of ChIP-seq datasets. qPCR validation of a range of ELK1 binding regions identified by ChIP-seq. Binding regions were ordered by descending tag count and candidates were selected by taking binding regions approximately every fiftieth and every one-hundredth position in the lists from the high and lower confidence ELK1 ChIP-seq datasets. ChIP-seq peak profiles for the ELK1 (blue vertical line) and IgG (yellow vertical line) samples are shown on the left. Bars in profile windows indicate 500 nt; RefSeq annotated genes are shown (if present) and the TSS is indicated by an arrow. The signal range (y-axis) is 0 to 100 for all regions apart from region 2 in the high confidence dataset (WNK1), where the maximum reaches 225. Specific binding of ELK1 to each region was validated in ChIP-qPCR experiments carried out in three biological repeats (shown on the right of the corresponding peak profiles); bars show the average percentages of input precipitated with the ELK1 antibody (blue bars) or non-specific IgG (yellow bars) with standard deviations. Titles of the graphs indicate the position of each region on a list ordered with descending tag count of ELK1 signal (prefix “L” denotes position on lower confidence list) and the name of the nearest annotated RefSeq gene. Binding regions from the lower confidence dataset are boxed. ABLIM2, CPLX1 and intron 3 of the SRF gene (SRFint3) are negative controls and EGR2 is a positive control. Data are summarized in Figure 2A. (PDF) [file pgen.1002694.s002.pdf]

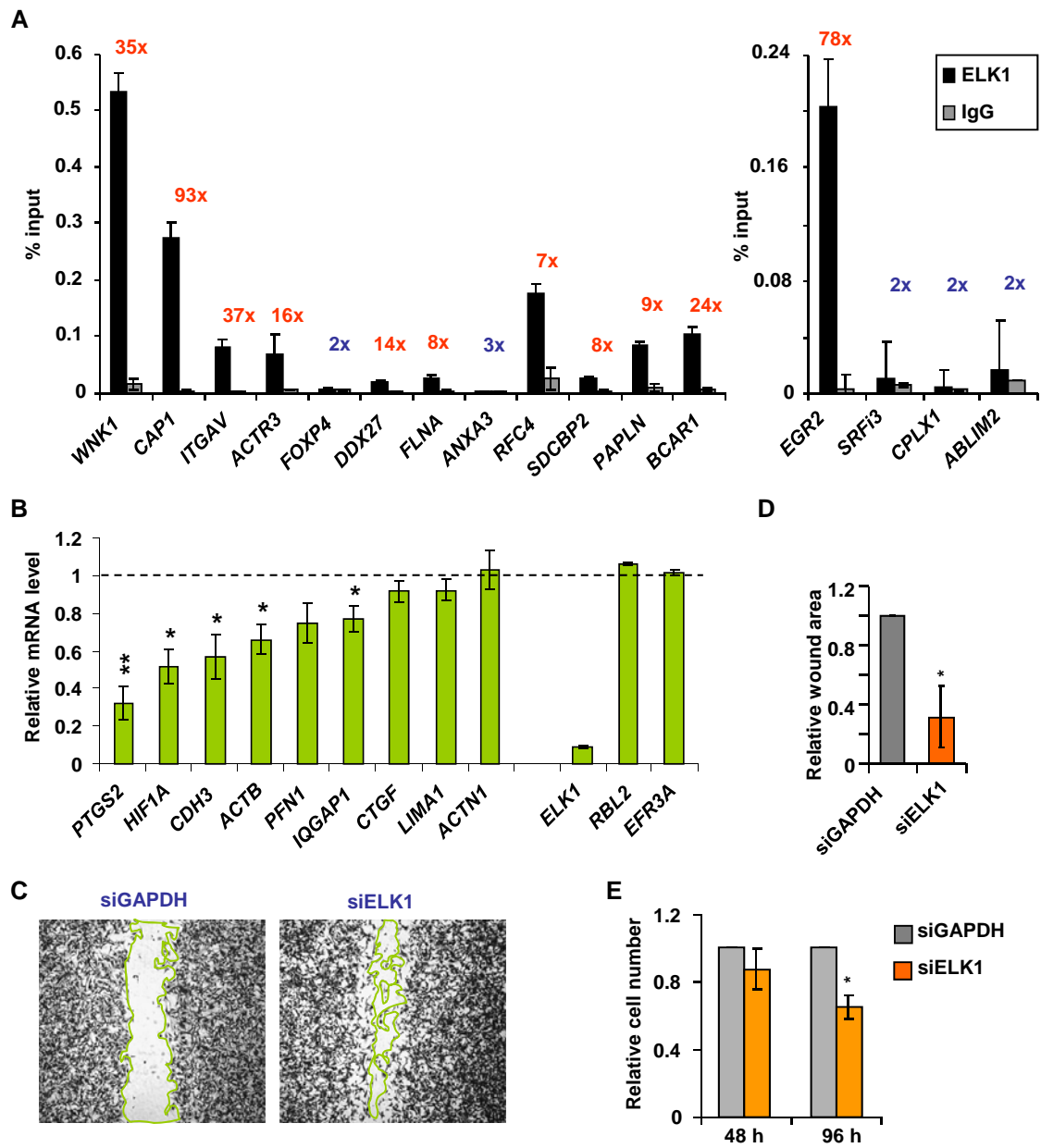

Supplementary Figure S3

Supplement: Figure S3 — Role of ELK1 in MDA-MB-231 cells. (A) ChIP-qPCR experiments were carried out in MDA-MB-231 cells for regions bound by ELK1 in MCF10A cells (see Figure 2A, and Figure S2; only the high confidence dataset was tested). Bars show the average percentages of input precipitated with the ELK1 antibody (black bars) or non-specific IgG (grey bars) with standard deviations. Numbers above bars show fold enrichment of ELK1 signal over IgG. EGR2 – positive control, ABLIM2, CPLX1, SRFint3 – negative controls. (B) The effect of depletion of ELK1 on mRNA levels of the indicated actin cytoskeleton- and migration-associated genes. RT-qPCR analysis of the mRNA levels of the indicated genes was carried out in serum starved MDA-MB-231 cells transfected with siELK1, with normalisation to an siGAPDH-transfected control; bars show average values from three biological repeats with standard deviations. Levels of ELK1 mRNA indicate the efficiency of depletion and RBL2 and EFR3A are control genes not associated with ELK1 Binding regions; * P<0.05, ** P<0.01 (Student's paired t-test). (C) Representative images of wounds created in monolayers of siGAPDH- or siELK1-transfected MDA-MB-231 cells, 18 hours post-stimulation with media containing 10% FBS. Lines show borders of areas which were used for quantification. (D) Areas of wounds in MDA-MB-231 cells treated as in (C) were measured in duplicates and normalised to control (siGAPDH). Bars show the average of three biological repeats with standard deviation. P-value was obtained from a two-tailed paired Student's t-test. (E) Numbers of MDA-MB-231 cells in a siELK1-transfected population were counted at 48 and 96 hours post-initial transfection and normalised to control (siGAPDH). Bars show average values of three biological repeats (performed in duplicates) with standard deviations. * P<0.05 (Student's paired t-test). (PDF) [file pgen.1002694.s003.pdf]

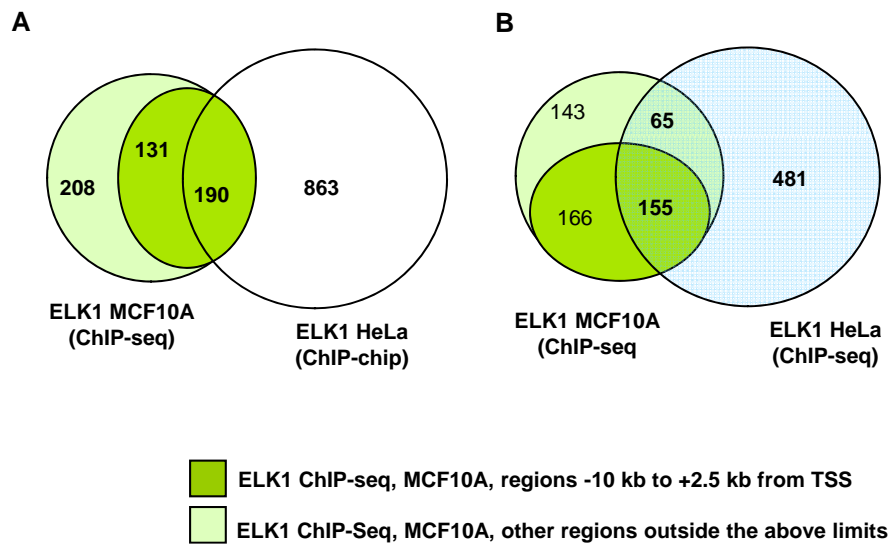

**Supplementary Figure S4**

Supplement: Figure S4 — Overlaps between ELK1 binding regions in MCF10A cells and HeLa cells. (A) Overlap between regions identified by ChIP-seq as bound by ELK1 in MCF10A cells, and by ChIP-chip as bound by ELK1 in HeLa cells [5]. (B) Overlap between regions identified by ChIP-seq as bound by ELK1 in MCF10A cells, and by ChIP-seq as bound by ELK1 in HeLa cells [17]. The ChIP-seq data from MCF10A cells is partitioned into promoter proximal (−10 kb to +2.5 kb from the TSS) and distal binding regions to enable comparison with the ChIP-chip data which only samples this region of the genome. (PDF) [file pgen.1002694.s004.pdf]

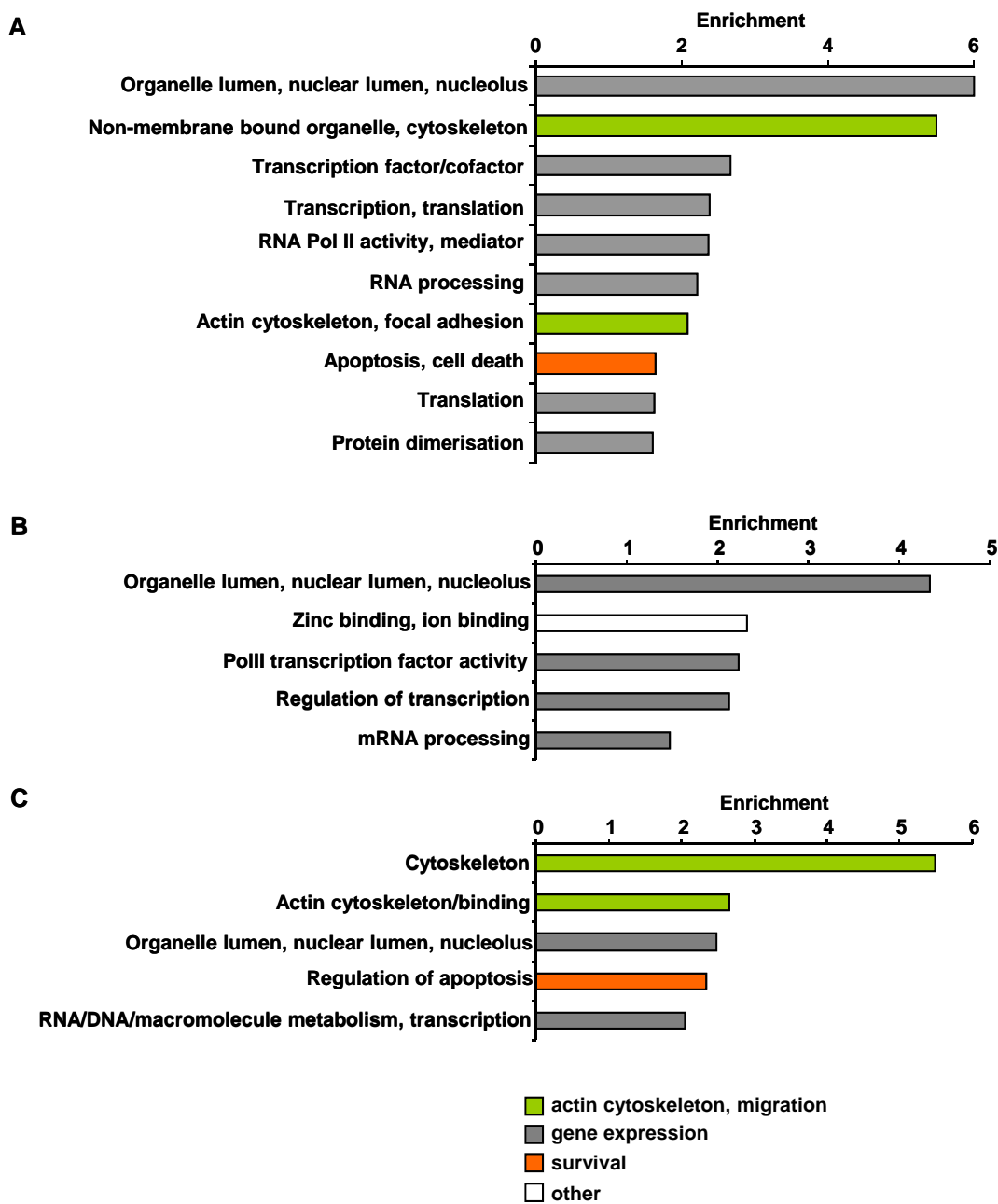

**Supplementary Figure S6**

Supplement: Figure S6 — Depletion of ELK1 changes the expression of distinct functional categories of genes. (A) DAVID functional clustering analysis was carried out for genes assigned to ELK1 ChIP-seq regions which show a significant change in expression levels in MCF10A cells depleted of ELK1 (as compared to an siGAPDH-transfected control), at either time point of EGF stimulation. Functional assignments of the top ten clusters are shown. GO terms were classified as gene expression-related (grey), actin cytoskeleton/migration-related (green) and cell survival related (orange). (B) DAVID functional clustering results for genes upregulated upon ELK1 depletion; the top five clusters are shown. (C) As in (B), but for downregulated genes. (PDF) [file pgen.1002694.s006.pdf]

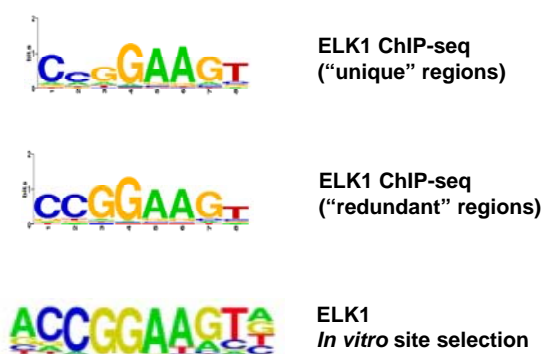

**Supplementary Figure S8**

Supplement: Figure S8 — Comparison of ELK1 binding motifs found in vivo and in vitro. TFBS logos obtained through STAMP-assisted visualisation of Weeder-derived position weight matrices of motifs overrepresented in “unique” and “redundant” ELK1-bound regions from the ChIP-seq experiment in MCF10A cells (see Figure 3C). For comparison, the logo for the ELK1 binding site obtained from an in vitro site-selection study [2] is shown at the bottom. (PDF) [file pgen.1002694.s008.pdf]

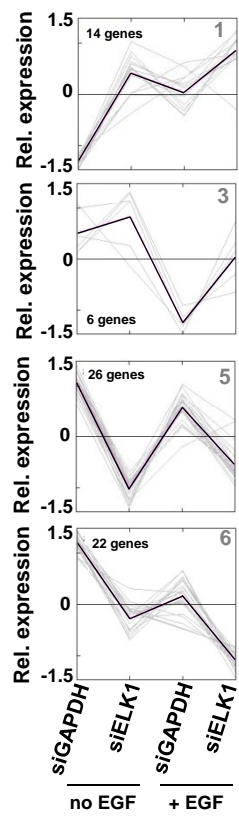

**Supplementary Figure S9**

Supplement: Figure S9 — “Unique” and “redundant” ELK1-bound regions regulate distinct sets of target genes. Summary profiles of the target genes in clusters 1, 3, 5 and 6 (see Figure 4A). The data are presented as changes of the individual (grey) and average (black) expression values of genes in each cluster under each of the four experimental conditions. For each gene, the mean of the signals across all four conditions was set as zero, and expression levels (z-transformed) are presented relative to this value. (PDF) [file pgen.1002694.s009.pdf]

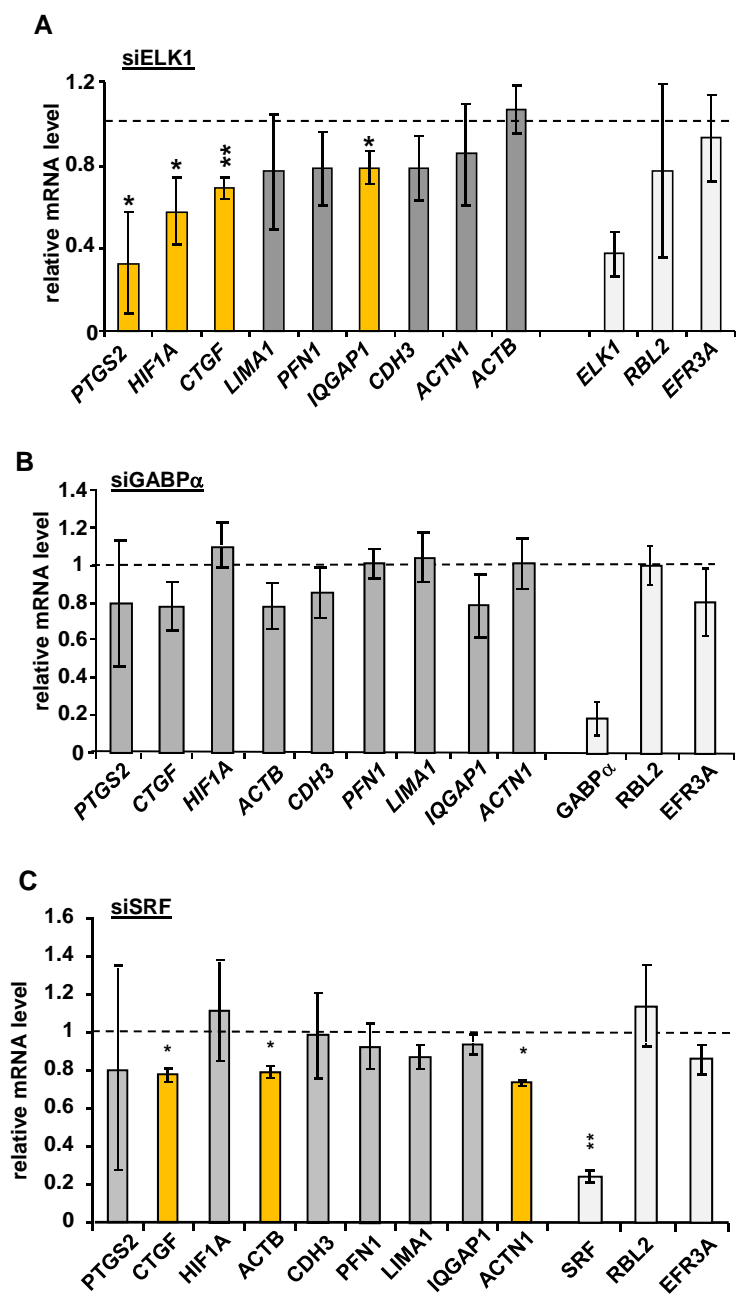

**Supplementary Figure S10**

Supplement: Figure S10 — The role of ELK1, GABPA and SRF in regulating “unique” ELK1 target genes. (A–C) mRNA levels of nine actin cytoskeleton- and migration-associated genes were measured by RT-qPCR from MCF10A cells grown in the absence of EGF (B and C) or additionally treated with EGF for 30 mins (A) and transfected with siRNAs against ELK1 (A), GABPA (B) or SRF (C); these were then normalised to an siGAPDH-transfected control (taken as 1). Bars show average values from two to three biological repeats with standard deviations. Levels of ELK1, GABPA and SRF mRNAs indicate the efficiency of depletion; RBL2, EFR3A are negative controls which do not associate with ELK1. Significantly altered expression is depicted with orange bars; * P<0.05, ** P<0.01 (Student's paired t-test). (PDF) [file pgen.1002694.s010.pdf]

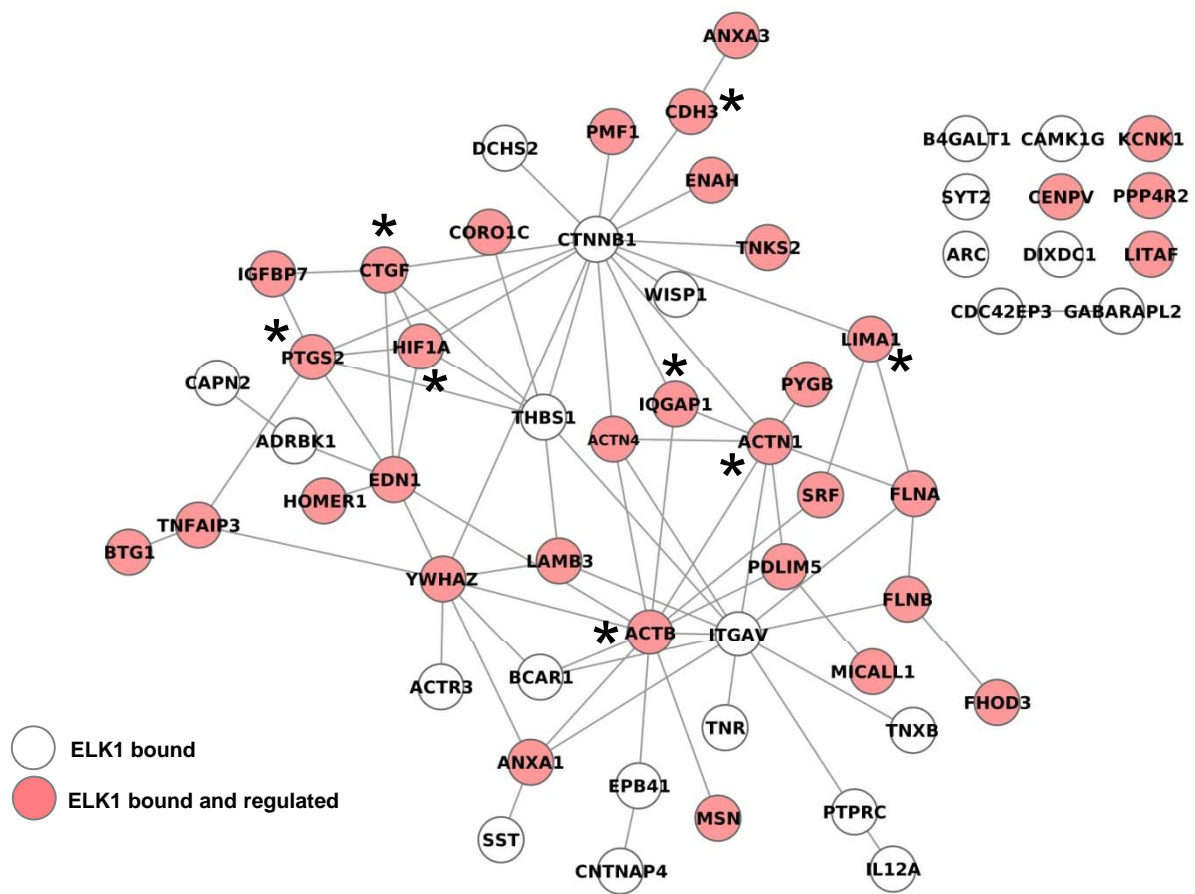

Supplementary Figure S11

Supplement: Figure S11 — ELK1 controls a network of actin/migration-related genes. Network formed by proteins encoded by actin cytoskeleton/migration-related genes associated with “unique” ELK1-bound regions (each protein denoted by a circle). Asterisks mark genes tested in Figure S10 and Figure 5A (PFN1 is associated with a redundant region). Pink circles indicate that gene expression is changed upon ELK1 depletion. Circles to the right of the figure show ELK1 target genes attributed to these functional terms but which do not have catalogued connections to the other genes in the network. (PDF) [file pgen.1002694.s011.pdf]

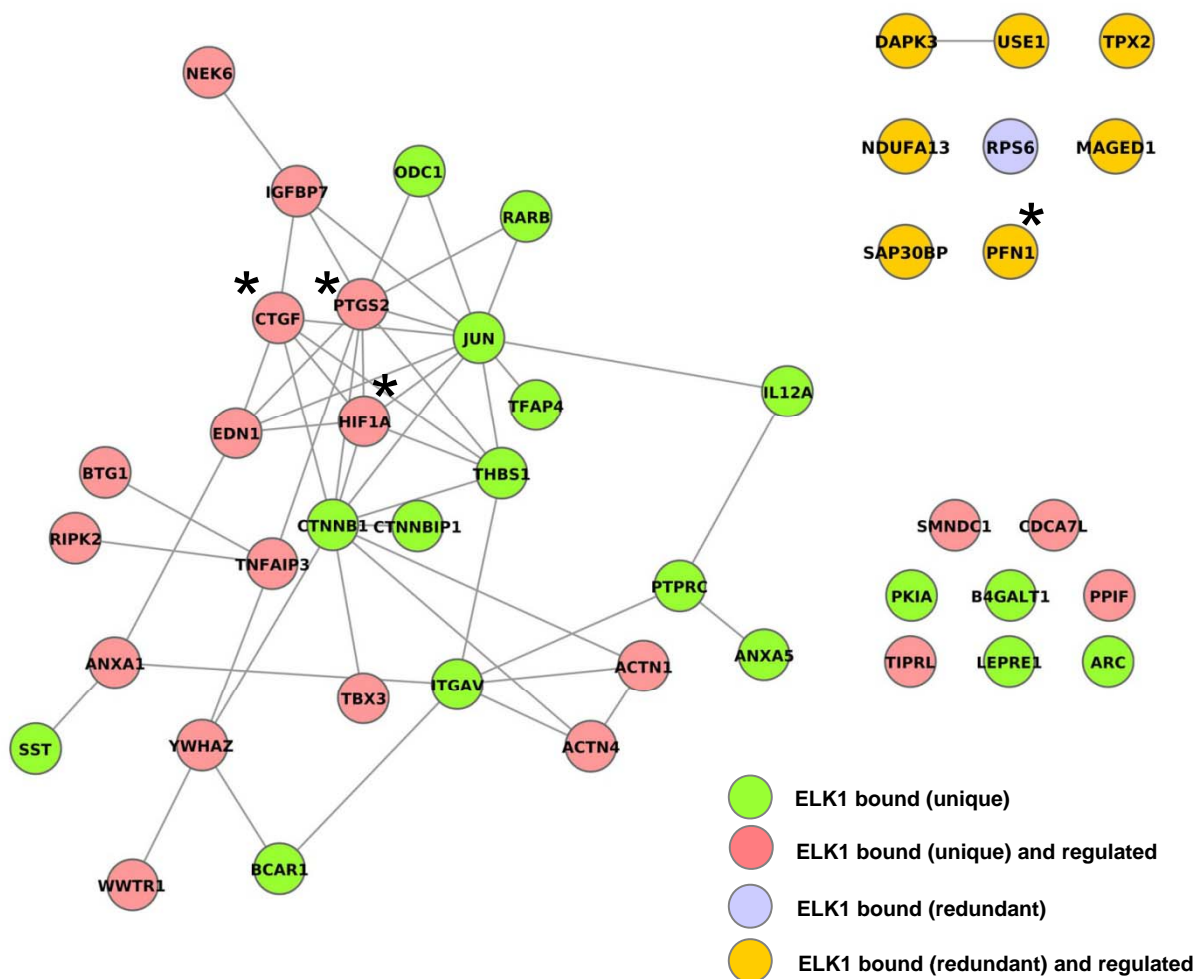

Supplementary Figure S12

Supplement: Figure S12 — ELK1 controls a network of survival-related genes. Networks formed by proteins encoded by survival-related genes associated with “unique” or “redundant” ELK1-bound regions (each protein denoted by a circle). Asterisks mark genes tested in Figure 5A and Figure S10, Pink and green circles indicate that the genes are associated with “unique” ELK1 binding regions and their gene expression is changed or unchanged respectively upon ELK1 depletion. Yellow and blue circles indicate that the genes are associated with “redundant” ELK1 binding regions and their gene expression is changed or unchanged respectively upon ELK1 depletion. Circles to the right of the figure show ELK1 target genes attributed to these functional terms but which do not have catalogued connections to the other genes in the network. (PDF) [file pgen.1002694.s012.pdf]

A

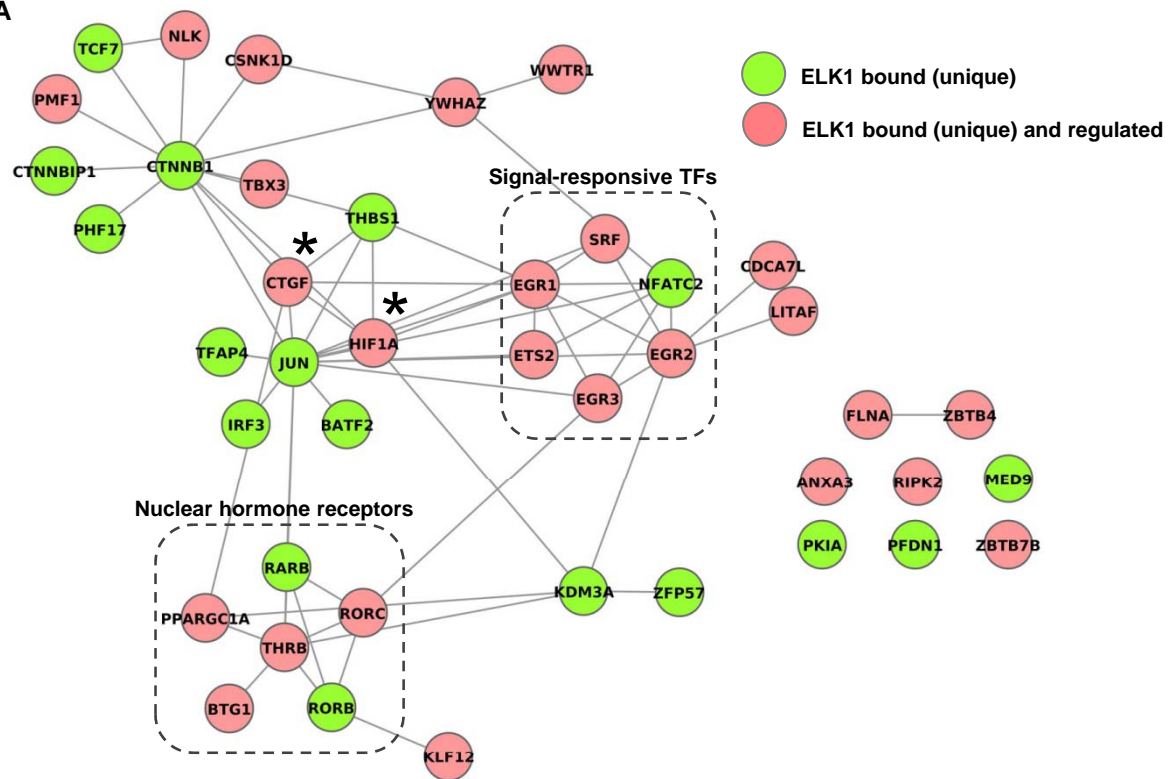

B

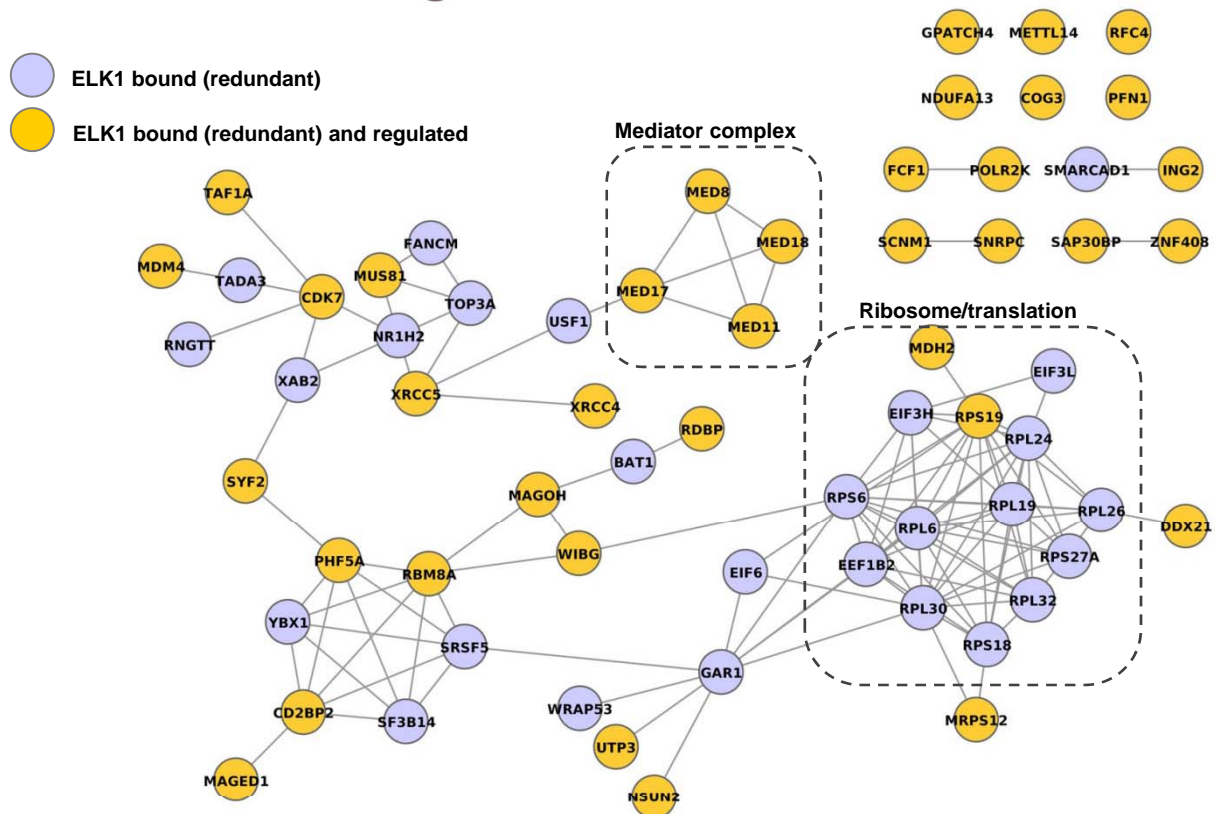

Supplementary Figure S13

Supplement: Figure S13 — ELK1 controls a network of gene expression-related genes. Networks formed by proteins encoded by gene expression-related genes associated with “unique” (A) and “redundant” (B) ELK1-bound regions. Asterisks mark genes tested in Figure S10, Figure 5A. Pink and yellow circles indicate that the genes are associated with “unique” and “redundant” ELK1 binding regions and their gene expression is changed upon ELK1 depletion whereas green and blue circles indicate that the genes are associated with “unique” and “redundant” ELK1 binding regions and their gene expression is unchanged upon ELK1 depletion. Circles to the right of the figure show ELK1 target genes attributed to these functional terms but which do not have catalogued connections to the other genes in the network. Subclusters of proteins that correspond to distinct categories of regulators are boxed. (PDF) [file pgen.1002694.s013.pdf]

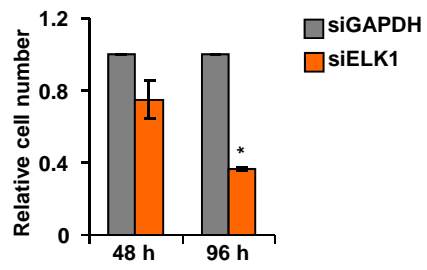

**Supplementary Figure S14**

Supplement: Figure S14 — Depletion of ELK1 impairs MCF10A cell survival. The numbers of MCF10A cells were determined 48 h and 96 h post-initial treatement with ELK1 siRNA and normalised to siGAPDH-transfected control. Cells were stimulated with EGF at t = 48 h (together with the second transfection). The experiment was performed in duplicate; average values and standard deviations from three biological repeats are shown. Significance was determined in a Student's paired t-test, * P<0.05. (PDF) [file pgen.1002694.s014.pdf]

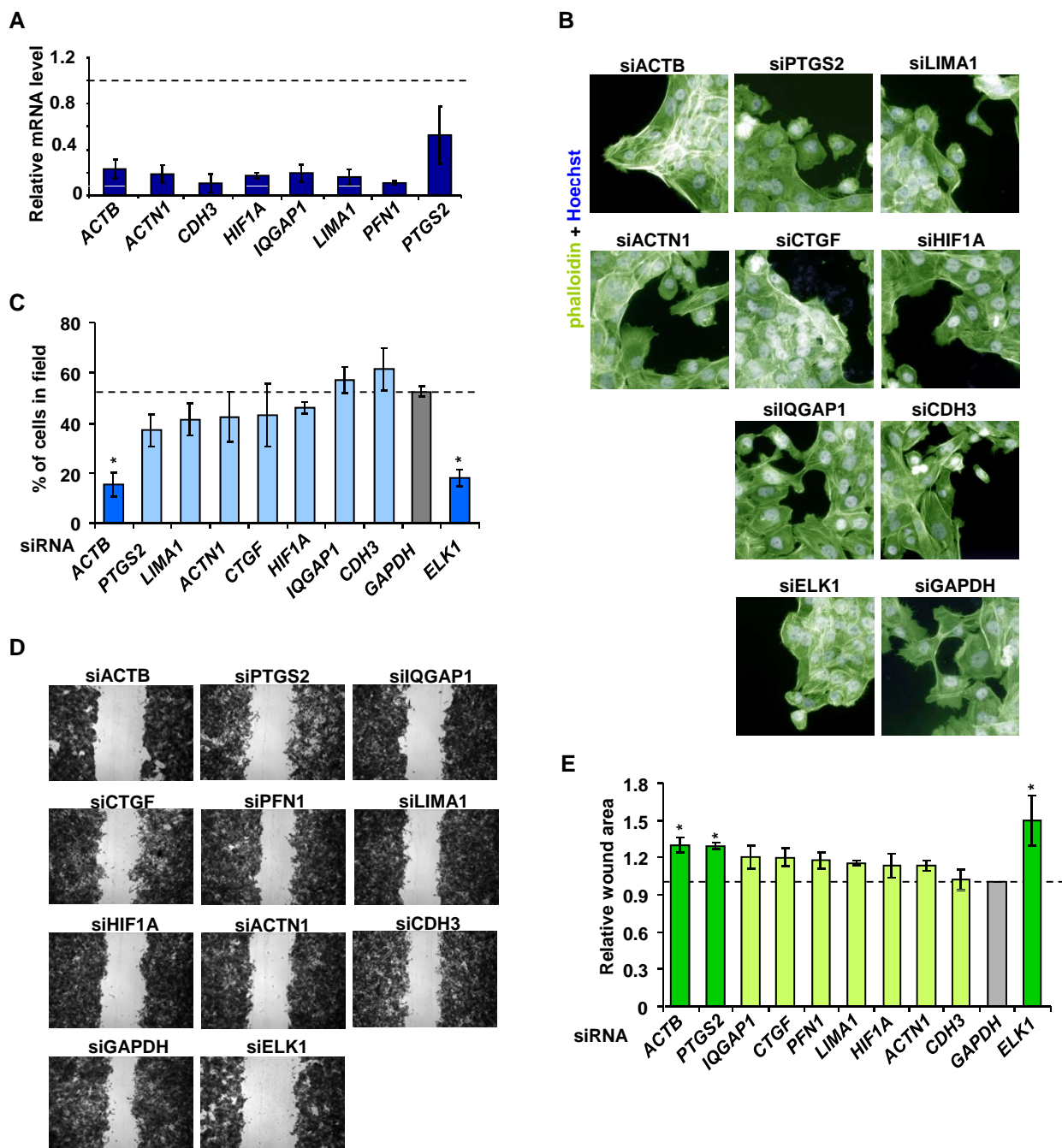

**Supplementary Figure S15**

Supplement: Figure S15 — ELK1-regulated genes control the actin cytoskeleton and motility of MCF10A cells. (A) Depletion levels of each of the indicated mRNA species following siRNA treatment were determined in RT-qPCR reactions. Bars show average values from three biological repeats with standard deviations, and are shown relative to cells treated with siGAPDH (taken as 1). (B) Representative images of MCF10A cells transfected with the indicated siRNA species, starved for EGF for 48 h and subsequently stimulated with EGF for 24 h and stained with phalloidin (green) and the Hoechst dye (blue). (C) The percentage of cells exhibiting membrane protrusions was calculated for each of the indicated siRNA transfections, as well as for siGAPDH (negative) and siELK1 (positive) – transfected controls. Bars show average values from three biological repeats with standard deviations; three fields were scored for each repeat. (D) Representative images of wounds created in monolayers of MCF10A cells transfected with the indicated siRNAs, are shown 15 hours post-stimulation with EGF (cells were stained with crystal violet). (E) Areas of wounds in MCF10A cells treated as in (D) were measured in duplicates and normalised to control (siGAPDH). Bars show average values of three biological repeats with standard deviations. P-values were calculated in Student's paired t-tests, * P<0.05 (PDF) [file pgen.1002694.s015.pdf]

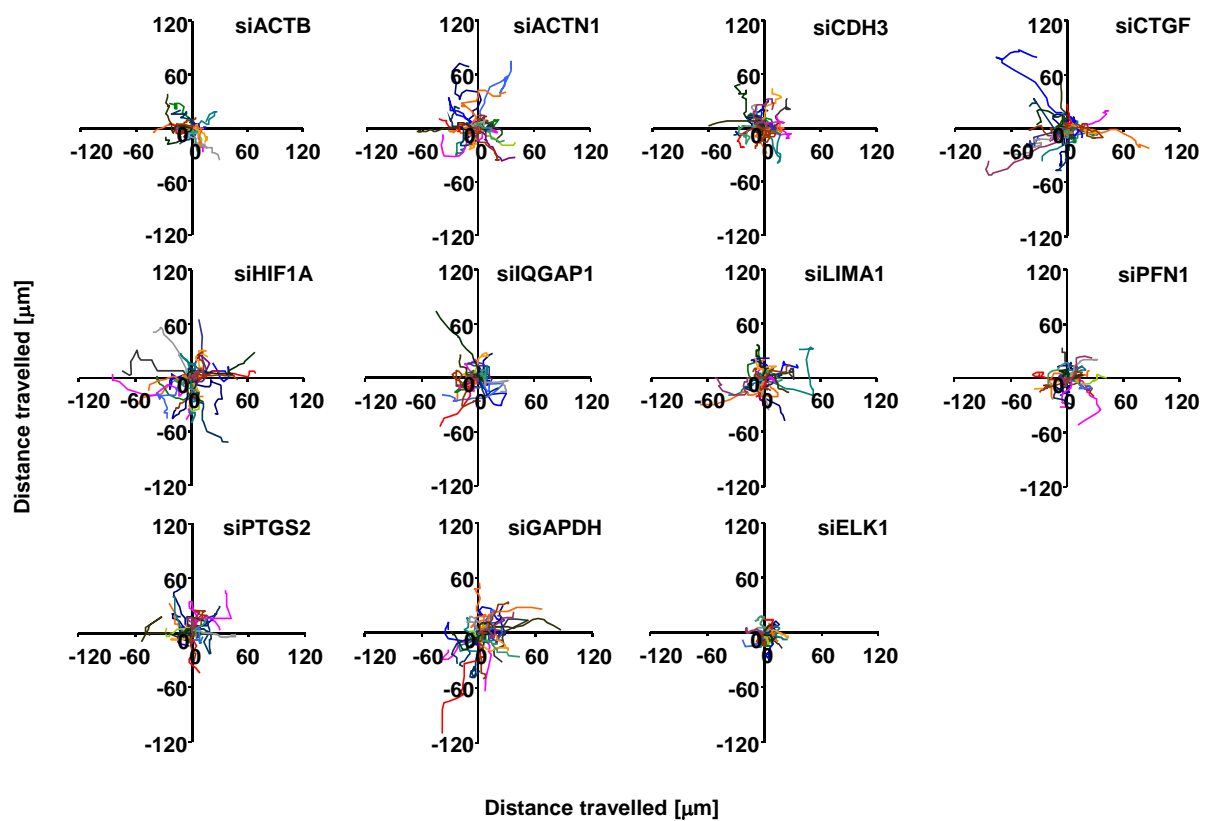

**Supplementary Figure S16**

Supplement: Figure S16 — Depletion of ELK1 target genes impairs MCF10A cell migration. Migratory trajectories of MCF10A cells transfected with the indicated mRNA species, manually tracked between t = 1 h and t = 7 h of EGF stimulation. Each coloured line represents the path travelled by an individual cell. (PDF) [file pgen.1002694.s016.pdf]
