# Supplementary material for: ELK1 Uses Different DNA Binding Modes to Regulate Functionally Distinct Classes of Target Genes
Source: PLoS Genet. 2012 May 10;8(5):e1002694. doi: 10.1371/journal.pgen.1002694 (PMC3349735; doi:10.1371/journal.pgen.1002694)
Supplement: Figure S5 — Gene Ontology analysis of regions bound by ELK1. GREAT Gene Ontology analysis was carried out for all regions from the high confidence dataset using default settings. Significantly enriched categories are shown. Categories associated with the actin cytoskeleton and migration are indicated by arrows. (PDF) [file pgen.1002694.s005.pdf]

Biological Process

Term Name	Binom Raw P-Value	Binom Fold Enrichment	Hyper Raw P-Value	Hyper Fold Enrichment
translation	**1.5192e-8**	3.0673	**3.8162e-5**	2.1268

Cellular Component

Term Name	Binom Raw P-Value	Binom Fold Enrichment	Hyper Raw P-Value	Hyper Fold Enrichment
ribosomal subunit	**1.6884e-7**	4.5653	**9.7923e-5**	2.8403
ribonucleoprotein complex	**4.4524e-7**	2.2713	**1.0783e-4**	1.7967
cytosolic part	**1.6981e-5**	3.1388	**7.9492e-5**	2.6972
exon-exon junction complex	**5.4757e-5**	42.6435	**3.7562e-4**	16.2897
nuclear body	**2.0226e-4**	2.4510	**4.2781e-4**	2.2580
actin cytoskeleton	**3.4671e-4**	2.0246	**1.1630e-4**	2.1208
nuclear speck	**4.4416e-4**	2.7863	**1.2437e-3**	2.5340
mediator complex	**1.1112e-3**	4.5006	**5.2378e-4**	4.7512

MSigDB Canonical Pathway

Term Name	Binom Raw P-Value	Binom Fold Enrichment	Hyper Raw P-Value	Hyper Fold Enrichment
Genes involved in Metabolism of proteins	**2.4391e-9**	4.1320	**2.3358e-5**	2.5023
Genes involved in Gene Expression	**3.7351e-9**	2.7267	**2.9901e-7**	2.2572
Spliceosome	**6.0691e-8**	4.8983	**2.0692e-5**	3.0784
Focal adhesion	**1.3132e-4**	2.2221	**1.5808e-5**	2.6195
JNK MAPK Pathway	**4.3699e-4**	3.7484	**2.7862e-4**	4.5725
Genes related to regulation of the actin cytoskeleton	**5.4008e-4**	5.1009	**1.8704e-4**	4.8266

Supplementary Figure S5
