# Supplementary material for: ELK1 Uses Different DNA Binding Modes to Regulate Functionally Distinct Classes of Target Genes
Source: PLoS Genet. 2012 May 10;8(5):e1002694. doi: 10.1371/journal.pgen.1002694 (PMC3349735; doi:10.1371/journal.pgen.1002694)
Supplement: Figure S7 — Overlaps between ELK1 binding regions and binding of other transcription factors; FOS/AP1 binding associates with “unique” ELK1-bound regions. (A) Overlap between regions identified by ChIP-seq as bound by ELK1 (MCF10A cells) and ELK4 (HeLa cells) [17]. (B) Overlap between regions identified by ChIP-seq as bound by ELK1 (MCF10A cells) and the indicated ETS transcription factors [2], [17], [24]. RPWE1 are normal prostate, Jurkat are T cell lymphoma, VCaP are prostate cancer, HL60 are leukaemia and HeLa are cervical cancer cells. The data are compared to the 303 “unique” and 226 “redundant” ELK1 binding regions, and the % overlap is presented (relative to the number of ELK1 regions). P-values are calculated in Chi square tests and significance values assigned to datasets which show a preferential differential enrichment with the “redundant” ELK1 binding regions. (C) Overlap between regions identified by ChIP-seq as bound by ELK1 (MCF10A cells), SRF (Jurkat cells; [19]) and GABPA (Jurkat cells; [19]). (D) Occurrence of FOS/AP1 motifs corresponding to the TGANTCA consensus (purple quadrants) in “unique” and “redundant” ELK1-bound regions. P-values were calculated using a Chi square test. (E) Western blot showing expression levels of FOS in MCF10A cells at indicated times after EGF addition. (F) Binding of FOS to “unique” (WNK1, ITGAV, PAPLN) and “redundant” (CAP1) regions was determined in MCF10A cells two hours post-EGF stimulation in ChIP-qPCR assays. Numbers above bars indicate fold enrichment of FOS signal over IgG. KLF9 – positive control. (PDF) [file pgen.1002694.s007.pdf]

A

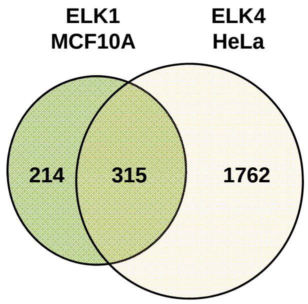

B

ChIP-seq	cell line	overlap "unique"	% 'unique'	overlap "redundant"	% 'redundant'	P-value
ETS1 Hollenhorst_2011	RWPE-1	3	0.99	41	18.14	P<1E-06
ETV4 Hollenhorst_2011	RWPE-1	19	6.27	6	2.65	N/S
ETV1 Hollenhorst_2011	RWPE-1	26	8.58	71	31.42	P=4.0E-06
ERG Hollenhorst_2011	RWPE-1	17	5.61	48	21.24	P<1E-06
ELF1 Wei_2010	Jurkat	22	7.26	173	76.55	P<1E-06
ERG Wei_2010	VCaP	22	7.26	57	25.22	P=1.24E-04
FLI1 Wei_2010	Jurkat	4	1.32	0	0.00	N/A
SPDEF Wei_2010	VCaP	15	4.95	28	12.39	P=1.954E-03
SPI1 Wei_2010	HL60	19	6.27	53	23.45	P<1E-06
ELK4 O'Geen_2011	HeLa	115	37.95	200	88.50	P<1E-06

C

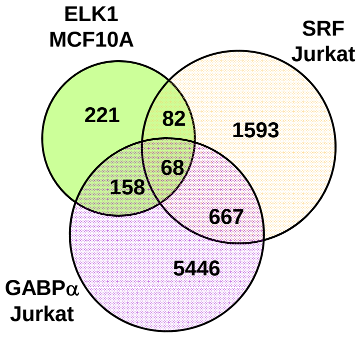

D

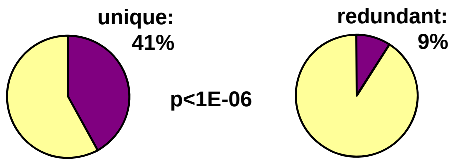

E

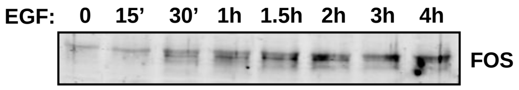

F

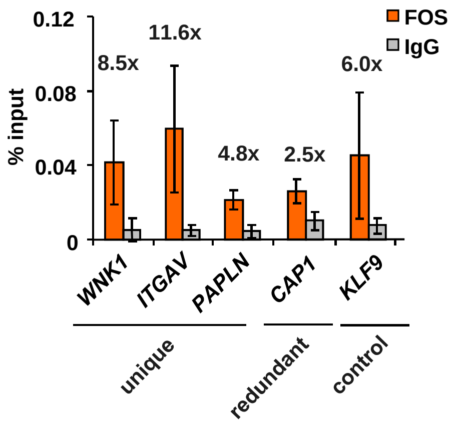

Supplementary Figure S7
