# Supplementary material for: ELK1 Uses Different DNA Binding Modes to Regulate Functionally Distinct Classes of Target Genes
Source: PLoS Genet. 2012 May 10;8(5):e1002694. doi: 10.1371/journal.pgen.1002694 (PMC3349735; doi:10.1371/journal.pgen.1002694)
Supplement: Table S5 — List of PCR primers used in (A) ChIP and (B) RT-PCR experiments. Nucleotide sequences are given in both cases, and the chromosomal locations of the primers used for ChIP (according to the hg18 genome assembly). (PDF) [file pgen.1002694.s021.pdf]

A. Oligonucleotide primers used in ChIP qPCR.

ASSOCIATED GENE	ADS NUMBER	SEQUENCE	REGION AMPLIFIED (Mar. 2006 (hg18) assembly)	PCR PRODUCT SIZE (bp)
WNK1	3111<br>3112	GCATGCCCGATTATTCTCTC<br>GCTCCTGGTTCTGCAGGTAG	chr12:703884-704102	219
CAP1	3113<br>3114	CAAGGAGTACGGAAGCGAAG<br>TCGGTGTGTTCTGTGACCAT	chr1:40278013-40278287	275
ITGAV	3115<br>3116	ATTCCCAGGAGCCTCTCTTT<br>CAGCCTGGCTTCCCTAAGTC	chr2:187148338-187148506	169
ACTR3	3117<br>3118	TTCGACTCCTGGTCATTTCC<br>CCAAGCACCGGTAATTCAGT	chr2:114364948-114365220	273
FOXP4	3119<br>3120	GCACAGATGACGAGGACAGA<br>CCTTCTAGGCGCATTCACTC	chr6:41612595-41612890	296
DDX27	3121<br>3122	ATACTGCCACCGTTTTGAGG<br>CTCGCCTATGGTTCCGATTA	chr20:47269136-47269431	296
FLNA	3123<br>3124	ACCTCCCCTCTCCAGAATGT<br>TAGACACCCAGGGCTACAGG	chrX:153254256-153254448	193
ANXA3	3125<br>3126	TTTGCATATTGAGGGTTGGA<br>AGGGTCTTGCTGTCAGTGCT	chr4:79692600-79692886	287
RFC4	3127<br>3128	AAGGACGAGATGGTCTCGAA<br>CCAGTTCTGCCTGAAAGTCC	chr3:188006839-188007109	271
SDCBP2	3129<br>3130	ACAAGCCACAAACTCCATCC<br>TGCGCTCTAGAAGTTGCTGA	chr20:1253748-1254001	254
PAPLN	3131<br>3132	TGGAGACACTGCTCCTGTCA<br>TGCCCCGATTCTGAATTTAT	chr14:72847960-72848083	124
BCAR1	3133<br>3134	GCTTTGGGGCTGAAAAACTA<br>GCAGCTCTGTGGTGTGTAGG	chr16:73836378-73836666	289
CAMKMT	3135<br>3136	TTGCTCTCTCAGCGTTTTTG<br>GTCAGGTGAAAGGGCTGACT	chr2:44827816-44828081	266
SLC13A4	3137<br>3138	TCCAAATGGAACATTGCTGA<br>GCCCATCAGGTAAAACAGGA	chr7:135042852-135043135	284
PSMB4	3139<br>3140	TCGCCACCTAGGATTACTGC<br>ACGGAATGCGGTAAAACTGT	chr1:149638537-149638763	227
ATF7IP	3141<br>3142	GGGAAGAACAGCCTGGTGTA<br>TGCTGGATTGTTCTGTCGTC	chr12:14318145-14318388	244
chip_*INT3 SRF*	1273<br>1274	GCCACAGGGCAGTAGATGTT<br>TCAGGCCCAAGTATCCACTC	chr6:43251941-43252121	181
EGR2	3343<br>3344	GTTCCGTTTTCGCTCCTGTA<br>TTTCTTGGCAGCTCAAAACC	chr10:64246622-64246805	184
ABLIM2	2599<br>2600	GGCTTGCTCAGAAACACTCC<br>ATCCTTCCTGAGTCCCCACT	chr4:8013205-8013391	187
CPLX1	2636<br>2637	GAACAGAGCAGCGCATATCA<br>CTGGGTCAGACCTGAGCTTC	chr4:766403-766527	125

B. Oligonucleotide primers used in RT-PCR

GENE	ADS NUMBER	STRAND	SEQUENCE
ACTB	3371<br>3372	FORWARD<br>REVERSE	TGACCCAGATCATGTTTGAG<br>CTTCATGAGGTAGTCAGTCAG
ACTN1	3373<br>3374	FORWARD<br>REVERSE	GCAGAGAAAGACATTCACGG<br>TCATCTTCACATTCCCATCCA
CDH3	3375<br>3376	FORWARD<br>REVERSE	GAAAGAAGCGGAAGATCAAGG<br>GATGTCATAGTCCTGGTCCTC
CTGF	2335<br>2336	FORWARD<br>REVERSE	AAGAGAACATTAAGAAGGGCA<br>GCGATTCAAAGATGTCATTGTC
HIF1A	3377<br>3378	FORWARD<br>REVERSE	CGTTGTGAGTGGTATTATTCAG<br>CAGGTTCCTTCTTAAGTTTGTC
IQGAP1	3379<br>3380	FORWARD<br>REVERSE	AAGTCCTGAACATAATCTCACC<br>GTATTCACATCTCCTGATTCCA
LIMA1	3381<br>3382	FORWARD<br>REVERSE	ATTCTCCAAGTACCAGAAAGCA<br>TGCTCTGTGCCTAATCTCAG
PFN1	3385<br>3386	FORWARD<br>REVERSE	AGACTGACAAGACGCTAGTC<br>GACAGACGAGGTCAGTACTG
PTGS2	3383<br>3384	FORWARD<br>REVERSE	TGTGTTGACATCCAGATCAC<br>GGAAGGGCTCTAGTATAATAGG
RBL2	2436<br>2437	FORWARD<br>REVERSE	CTGAACTTATGATGGACAGAC<br>ACACTTCTATACACCTGGCTC
EFR3A	3363<br>3364	FORWARD<br>REVERSE	GGAGGACTACGAACTGAGAC<br>ACATCCGGTATTATTCTGATCCC
ELK1	2113<br>2114	FORWARD<br>REVERSE	GGTGGTGAATTCAAGCTGGT<br>ATTTGGCATGGTGGAGGTAA
GABPA	2442<br>2443	FORWARD<br>REVERSE	AATCTGCTACACCTACTACCA<br>CATTGTTTCCTGTTCTGTTCC
SAP1	2276<br>2277	FORWARD<br>REVERSE	TCGCAAGAACAAGCCTAACA<br>CTTTCCCTCCATTCTCCACA
KLHL28	3365<br>3366	FORWARD<br>REVERSE	GTGGTGGGTGGACATAATGGAGTC<br>CGATTACAGCAGCACCAACTCCTG
MDM4	3367<br>3368	FORWARD<br>REVERSE	CTTCTCCGTGAAAGACCCAAGC<br>ATACTGTGATCCTGTGCGAGAGC
SCNM1	3369<br>3370	FORWARD<br>REVERSE	AGGAGTCAGCAACTGTCTCAGC<br>CATCTGGGATCCATCCAGAGCTTC

Table S5
